# Game-Calibrated and User-Tailored Remote Detection of Stress and Boredom in Games

**DOI:** 10.3390/s19132877

**Published:** 2019-06-28

**Authors:** Fernando Bevilacqua, Henrik Engström, Per Backlund

**Affiliations:** 1 Computer Science, Federal University of Fronteira Sul, Chapecó 89802 112, Brazil; 2 School of Informatics, University of Skövde, 541 28 Skövde, Sweden

**Keywords:** human–computer interaction, games, affective computing, remote photoplethysmography

## Abstract

Emotion detection based on computer vision and remote extraction of user signals commonly rely on stimuli where users have a passive role with limited possibilities for interaction or emotional involvement, e.g., images and videos. Predictive models are also trained on a group level, which potentially excludes or dilutes key individualities of users. We present a non-obtrusive, multifactorial, user-tailored emotion detection method based on remotely estimated psychophysiological signals. A neural network learns the emotional profile of a user during the interaction with calibration games, a novel game-based emotion elicitation material designed to induce emotions while accounting for particularities of individuals. We evaluate our method in two experiments (n=20 and n=62) with mean classification accuracy of 61.6%, which is statistically significantly better than chance-level classification. Our approach and its evaluation present unique circumstances: our model is trained on one dataset (calibration games) and tested on another (evaluation game), while preserving the natural behavior of subjects and using remote acquisition of signals. Results of this study suggest our method is feasible and an initiative to move away from questionnaires and physical sensors into a non-obtrusive, remote-based solution for detecting emotions in a context involving more naturalistic user behavior and games.

## 1. Introduction

The process of detecting user emotions, an important element of affective computing [1], has wide applications in the field of human–computer interaction (HCI), particularly in games research. One of most the common techniques used to obtain data regarding the emotional state of players in a game is psychophysiological measurements [2]. Physical sensors provide uninterrupted measurement of user’s physiological signals without affecting the task at hand [3,4,5,6]. A significant amount of psychophysiological information can be read from the human body, including signals such as heart rate (HR), respiratory rate (RR), facial actions (FA), among others. The combined analysis of those different signals, known as multimodal or multifactorial analysis, is more likely to produce accurate results in the mapping of signals into emotional states [7,8]. The use of physical sensors, despite avoiding interruptions, is usually perceived as uncomfortable and intrusive, since they need to be properly attached to various parts of the user’s body. Additionally, sensors might restrict player’s motion abilities, e.g., a sensor attached to a finger prevents the use of that finger. Sensors also increase the user’s awareness of being monitored [9,10,11], which affects the results of any emotional modeling.

The use of remote sensing to acquire psychophysiological signals from users, a non-obtrusive data collection approach, is mentioned in the literature as a possible solution for this problem. Facial analysis, for instance, is an unobtrusive approach for emotion detection of players [12]. Advances in computer vision allow detailed inspection of facial activity, making automated facial analysis enough to infer emotions of players [13,14]. In that sense, automatically detected facial expressions have been used in the contexts of games, including to correlate dimensions of experience [15] and to enhance online games [16,17]. Another common signal used in emotion detection is HR, which can also be acquired using remote, non-obtrusive approaches. The use of remote photoplethysmography (rPPG) allows the estimation of HR and heart rate variability (HRV) using ordinary RGB cameras [18]. Physiological arousal is connected to emotion regulation [19,20,21], in particular the interactions of the Autonomic Nervous System (ANS). Consequently, physiological signals such as the HR are considered reliable sources of information due to their link to the ANS, as opposed to facial expressions [22], for instance. Despite the potential rPPG has to estimate HR without physical sensors, the natural behavior of users in a gaming context, e.g., movement and facial expressions, considerably impacts estimations [23]. Although these challenges are limiting factors, the use of rPPG has already been applied to emotion detection. Signals, such as HR and HRV, were used to remotely detect stress [3,24,25], for instance. In the majority of the cases, however, subjects are instructed to stay still [18], which leads to improved accuracy in the estimations. Such behavioral constraints affect the interaction between players and games making the experience unnatural.

The process of mapping physically or remotely acquired psychophysiological signals, e.g., HR and FA, into emotional states involves the identification of the signals that better predict the emotional states [26], as well as a proper definition of the emotional states themselves [27]. A common approach used to perform such mapping is the application of machine learning. The use of machine learning commonly starts with a group of users being exposed to emotion elicitation materials, e.g., images and videos with known emotional labels such as stress and boredom. Signals from those users, e.g., HR and facial expressions, are then measured during the interaction and used to train a machine learning model according to the labeled elicitation materials. Consequentially, predictive models are based on a collective perspective. Since a model is usually trained from data of several users, in practice, it potentially describes the average behavior of the group and excludes the key individualities of each user. Such individualities are the main characteristics that define a person, since people are not similar in several aspects such as play and learning styles [28], as well as different responses to media based on personality [29]. It has been suggested that a user-tailored approach, where a model is trained from a single individual instead of a group, is more likely to produce better emotional estimations [30].

In summary, previous work focused on obtrusive, physical sensors to acquire user signals for emotion detection. Even when non-obtrusive approaches are used, e.g., rPPG and facial analysis based on computer vision, models are trained based on emotion elicitation materials with limited interactivity, e.g., images and videos. When exposed to such materials, subjects face a passive role with limited opportunities for interaction. Additionally, such materials do not adapt themselves to account for differences in cultural expectations of subjects, assuming all subjects experience the pre-defined emotional stimuli in the same way. Hence, it is reasonable to believe that individual characteristics of users might be better observed with more personalized and complex emotion elicitation materials such as games. Furthermore, a user-tailored approach is likely to preserve and better account for individual characteristics in a method for emotion detection, as opposed to a group model to detect emotions. Additionally, models created from a group are highly affected by ethnic and gender bias, since it is significantly difficult to obtain data from a group that accurately represents the world population. Such limitation is considerably mitigated in a user-tailored model, since the approach is, by design, based on the data of a single person who is already a perfect representation of him/herself.

**Figure 1:**
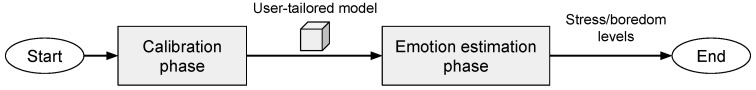
General structure of our proposed method for remote detection of stress and boredom of players during their interaction with games.

In that light, there is a lack of initiatives focusing on non-obtrusive, user-tailored emotion detection models within the context of games research that is based on game stimuli. This paper presents an approach that aims to fill that gap, providing the HCI and games research community with a process to remotely detect the emotional state of users in a non-obtrusive way. Our approach uses remotely acquired signals, namely HR and FA, to create a user-tailored model, i.e., trained neural network, able to detect emotional states of boredom and stress of a given subject. The approach is composed of two phases: training (or calibration) and testing, as illustrated in Figure 1. In the training phase, the model is trained using a user-tailored approach, i.e., data from subject $S_{a}$ playing calibration games are used to create model $N_{a}$. The calibration games are a novel emotion elicitation material introduced by this research. These games are carefully designed to present a difficulty level that constantly progresses over time without a pre-defined stopping point, inducing emotional states of boredom and stress, which accounts for the individualities of each user. The result of the training phase is a user-tailored model, i.e., model $N_{a}$, which is a trained neural network aimed for use on subject $S_{a}$. Finally, the testing phase is conducted in a game session involving subject $S_{a}$ playing any ordinary, non-calibration game, e.g., Super Mario. During the testing phase, the signals of subject’s $S_{a}$ are remotely acquired and fed into the previously trained model $N_{a}$, which then outputs the estimated emotional state of subject $S_{a}$ for that particular testing game. Subjects are not instructed on how to behave during the process, which makes the method more generalizable to a context where users behave and interact with games naturally. Our main contribution is a method for detecting emotional states of stress and boredom of users during gaming sessions using remote acquisition of signals via computer vision, a user-tailored model and emotion elicitation based on a novel game-based calibration phase. The approach is automated and implemented as a software without the need of specialized equipment, e.g., sensors. Only a regular video camera and a computer are needed. We present the conception, feasibility test, and systematic evaluation of our method conducted to test the overarching hypothesis: an emotion detection approach that uses remotely acquired signals, i.e., HR and FA, as input of a machine learning model, i.e., neural network that is trained on a user-tailored basis (one subject produces one model) using calibration games as emotion elicitation can achieve an accuracy rate better than chance-level classification. Results are derived from two different experiments with a heterogeneous group of subjects, i.e., n=20 and n=62, who behaved naturally during the interaction with the games. Previous studies use physical sensors, non-interactive emotion elicitation materials, e.g., images, instructions for subjects to keep still, and highly correlated samples for training and testing any emotion classification model. Our approach and analysis, however, features unique circumstances: our user-tailored model is trained on one dataset (calibration games) and tested on another (evaluation game), while preserving the natural behavior of subjects and using remote acquisition of signals. This configuration is significantly more challenging than previous work. Our proposed method has use cases in both the academia and industry. Regarding the former, researchers of games and HCI can use our approach to detect the emotional state of users during the interaction with digital games and information systems non-obtrusively and without interruptions, e.g., answering questionnaires. The detection of stress and boredom, for instance, is directly related to the engagement level of users as represented by the Theory of Flow [31]. In the industry, the unobtrusive detection of stress and boredom levels can be used to evaluate games, since said emotions are key components in the challenge–reward cycle that makes a game fun and engaging [32]. An evaluation regarding stress and boredom, for instance, allows game developers and publishers to optimize their products before market, leading to higher chances of financial success. Finally, game developers can explore new ways of creating games that detect stress and boredom levels without additional hardware or sensors, making games self-adjust the difficulty level during run-time to improve players’ experience.

## 2. Remote Detection of Stress and Boredom

**Figure 2:**
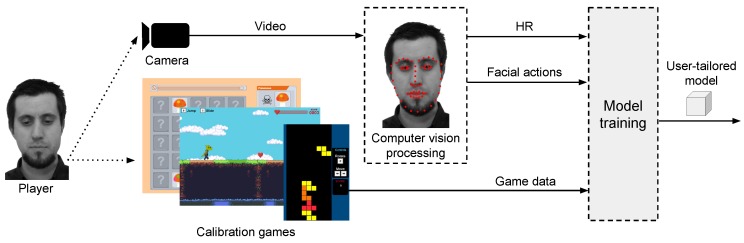
Calibration phase composed of emotion elicitation games (calibration games) and remote acquisition of signals from the user. The result of this phase is a user-tailored model applied to detect emotions.

The general structure of our proposed method for remote detection of emotions contains two main phases, as previously mentioned: a calibration and an emotion estimation phase. In the calibration phase, the user plays a set of carefully designed games, named calibration games that act as emotion elicitation materials. During this phase, illustrated in Figure 2, user signals elicited from the interaction with the games, e.g., HR and facial actions, are remotely acquired and used to train a user-tailored model, i.e., neural network. This model is the foundation for the detection of emotions of that particular user. Finally, in the emotion estimation phase, the user interacts with any ordinary game, e.g., commercial off-the-shelf (COTS) game, while his/her signals are remotely acquired and fed into the previously trained user-tailored model. The model then outputs the emotional state of stress and boredom for that user in that game.

As a consequence of the design of the calibration games (detailed in Section 2.1), which induce particular emotional states, the remotely collected information from the user during the calibration phase contains a detailed variation profile of that user, including changes of psychophysiological signals, throughout the theoretically known emotional states. If a person has a better response to a certain physiological signal instead of another, e.g., HR over facial actions, then the variation of that signal is likely to be considered more relevant by the machine learning model during its training. Since the training process is completely based on the signals of a single user, nuances and individual behavior are likely to be registered and learned. The calibration phase needs to be performed once per person.

**Figure 3:**
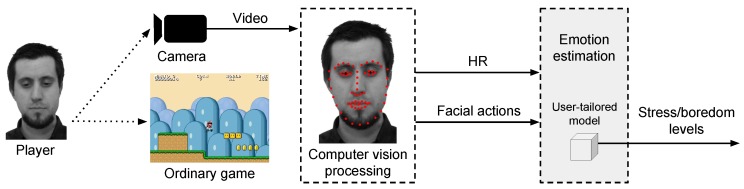
Emotion estimation phase. Remotely acquired signals from the player are fed into a user-tailored model that outputs the stress/boredom levels of the player during the interaction with an ordinary game.

After the calibration phase, the person can play any other ordinary game and be monitored in an emotion estimation phase, as illustrated by Figure 3. As the user plays the game, signals are remotely acquired based on the analysis of the video feed of the interaction. These signals are then used as input to the previously trained user-tailored model of that particular person, which, as a result, produces an estimation of the emotional state regarding stress and boredom for that person in that game. Given that a user has a trained user-tailored model, the emotion estimation phase can be performed for any game as many times as desired.

### 2.1. Game-Based Emotion Elicitation

Different types of emotion elicitation materials have been mentioned in the literature regarding emotional stimuli. Commonly, emotion elicitation materials are based on images [33,34], videos [30,35] and gamified cognitive tests [24,36]. The use of computer games as emotion elicitation material is less common; however, they have been proved to provoke alteration in the mean HR of players at stressful periods of gameplay [37,38]. The type of emotion elicitation material also impacts the behavior of subjects. When images and videos are used, for instance, subjects take a passive role with limited possibilities of interaction, more likely resulting in less emotional and corporal manifestations. If games are used instead, users can take an active role in the interaction and are more likely to behave in a natural way, e.g., featuring facial expressions and moving the body [39]. In a game, users present some degree of body movement, e.g., move of fingers in regard to controllers. Users are also in charge of actions related to the mechanic, whose consequences, e.g., wrong/right moves, is likely to produce an emotional reaction associated with movement, e.g., laugher and head tilt. Therefore, it is plausible to believe that games present a more sophisticated interaction through their mechanics, as opposed to the simplistic, one-way interaction between users and images/videos, for instance. Consequentially, detecting emotions of users during their interaction with games presents a set of challenges inherently associated with the natural behavior of users in that context. As such, the use of games as emotion elicitation materials is likely to create an emotional connection between users and the stimuli while still portraying the expected natural behavior existent in a gaming session, resulting in clear and observable changes in body language and psychophysiological signals.

**Figure 4:**
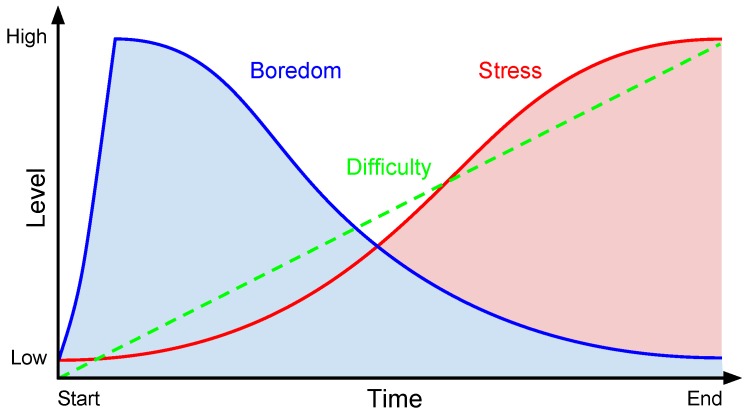
Structure of a calibration game. The *x*-axis shows the progression of time. The *y*-axis shows the game difficulty and the emotional state users should experience, i.e., stress and boredom.

Part of the novelty of our approach relies on the use of games as emotion elicitation materials, which are likely to induce richer emotional reactions, as previously mentioned. In the calibration phase of our method, three games are used to provoke emotional changes in the subjects, so an emotional profile can be learned. We call them calibration games because their goal is to induce an emotional state of boredom and stress on the subject on a user-tailored basis, accounting for different gaming skills and expectations, as illustrated by Figure 4. At the beginning, the difficulty level (green line) is low and the games are highly predictive and without changes or surprises with a focus on the passage of time and waiting. Such characteristics lead the user to an emotional state of boredom (blue curve) [32,40,41]. The games’ difficulty is then constantly and periodically adjusted, which makes them more challenging as time progresses. At some point in time, which is different for each user depending on gaming skills and personal preferences, the pace of the gameplay and the difficulty level will be overwhelming, leading the user to an emotional state of stress (red curve) [32,39]. As the difficulty level continues to increase, the stress level of the user will also increase. Finally, the difficulty level will increase to the point at which the user is unable to cope with the game. This will lead to consecutive mistakes in the game that will eventually terminate it, e.g., health bar of the main character reaches zero. Consequently, the calibration games should account for the different expectations and gaming skills of a wide range of users while inducing boredom and stress. Three calibration games were developed for the experiments presented in this paper. They were 2D and casual-themed, and played with a mouse or keyboard in a web browser. The game mechanics were chosen to prevent the subject to control the pace of the game, which ensures that all the subjects have the same game pace. As previously described, the difficulty of the games increases constantly and the pace is used as a main element to induce particular emotions. Additionally, the mechanics were selected to prevent players from instantly killing the main character by mistake, avoiding an abrupt termination of the interaction.

Previous analysis [39] conducted on the three calibration games mentioned in this paper, i.e., *Mushroom*, *Platformer* and *Tetris* (see Section 4.1), show that players’ self-reported emotional state indicate they perceived the games as being boring at the beginning and stressful at the end. This is an essential characteristic of our idea of calibration games, since they are expected to induce emotional states of boredom and stress, as previously mentioned. It has also been confirmed that at boring and stressful parts of such calibration games players present different mean HR [42] and facial activity [39,43]. This strengthens the idea of our calibration games being used as emotion elicitations materials able to evoke psychophysiological reactions that can be used to train a machine learning model for emotion detection.

### 2.2. Remote Extraction of User Signals and Classification Features

The classification efficiency of a machine learning model is related to the number of features able to accurately discriminate the elements being classified, and the use of more features does not necessarily produce a better model [44] (Chapter 6). Hence, we use a set of features for classification derived from psychophysiological signals that are remotely extracted from a video. They were designed on the basis of previous reports regarding the potential of said signals and features to differentiate emotional states in games. In total, eight features, denoted $F_{1}$ to $F_{8}$, are calculated: $F_{1}$ to $F_{7}$ are related to facial activity, while $F_{8}$ is related to HR activity estimated using rPPG. Table 1 presents a description of all the features.

Features $F_{1}$ to $F_{7}$ are based on 68 facial landmarks automatically detected using Constrained Local Neural Fields (CLNF) [46,47]. They are mostly calculated using the Euclidian distance between those facial landmarks, and they are modeled after facial elements that express a connection with emotional states (see Bevilacqua et al. [43] for a detailed description of the features). This connection is mentioned in Section 3, such as activities of the corrugator, the zygomatic and the orbicularis oculi muscles which are related to positive game events [48], positive emotions [6], and rewarding game events [49], respectively. The detection of stress is also related to blinking rate [33,50], lip movement [50] and lips deformation [33,51], mouth activity [52], and head movement/velocity [33].

Feature $F_{8}$ is based on remote estimations of HR performed using an established rPPG technique, i.e., Poh et al. [45]. As mentioned in Section 3, HR and its derivatives are well founded sources of information used for emotion detection [7]. Reports in the literature show the use of HR and its derivatives for continuous arousal monitoring [35], the measurement of confusion [53], the detection of emotional reactions related to digital media [54], the detection of mental and physical stress [55,56], and the measurement of frustration [38]. The rPPG technique proposed by Poh et al. [45] has been reported in the literature as significantly resilient when estimating the HR under challenging conditions, thus we have selected such technique as a suitable way to estimate the HR of users within a context involving games and the natural behavior of players.

The process of extracting and calculating the features is based on a moving window applied to the input video. The moving window has a size of 15 s and a step of one second (93.33% overlap). For each window in the video, the previously mentioned CLNF and rPPG techniques are applied to all frames within that window, to detect facial landmarks and collect information regarding pixel values, e.g., the mean value of pixels in the blue channel. Even though all frames within the window are analyzed, only a single, final value is assigned to each feature per window. For features $F_{1}$ to $F_{7}$, the final value of a given feature is calculated by aggregating the values of all the frames within the window of that given feature by using mean or standard deviation. Empirical tests conducted for this research have shown that features connected to facial regions with fast changes within the window, e.g., eye area and face motion, are better represented by an aggregation using the standard deviation. However, facial features with slower changes, e.g., face area and mouth activity, are better represented by an aggregation using the mean. Consequentially, features $F_{1}$, $F_{2}$ and $F_{5}$ are aggregated using the mean, while features $F_{3}$ and $F_{4}$ are aggregated using the standard deviation. Feature $F_{8}$ does not require any aggregation of values, since all frames within the window are used to produce a single value, i.e., the estimation of the mean HR in that window.

## 3. Background and Related Work

An important aspect of emotion detection relies on physiological signals being linked to emotion regulation [19,20,21]. The ANS, which is related to physiological arousal [19], is subdivided into the Sympathetic and Parasympathetic Nervous System (SNS and PNS, respectively). These systems interact (often antagonistically) to produce variations in physiological signals to prepare a person to react to a situation. The SNS is dominant during physical and psychological stress, triggering the body to alertness, by increasing the HR, for instance. The PNS, on the other hand, is dominant in periods of stability and relative safety, maintaining physiological signals at a lower degree of arousal, e.g., by decreasing the HR. The continuous changes between the SNS and PNS impulses cause, for instance, variations of HR and HRV [21], which refers to beat-to-beat alternations in HR intervals.

Physiological signals are interesting for emotion detection because suppressing emotions or social masking through physiological signals is impossible [57]. In the games research field, the use of physiological signals to automatically assess different emotional states has been demonstrated [3,4,5,6]. Commonly, the process of emotion detection involves three main steps: emotion elicitation stimuli, feature extraction, and classification. In that configuration, subjects are exposed to emotion elicitation materials which induce changes in psychophysiological signals. The signals are then physically or remotely read and a set of features is derived from them. Finally, the features are used to train a machine learning model able to classify emotions based on the patterns of changes of said signals/features.

Different approaches exist for emotion elicitation stimuli, feature extraction, and classification methodologies regarding emotion detection. The following sections present works and theories related to each one of those elements.

### 3.1. Extraction and Selection of Psychophysiological Signals

The extraction of psychophysiological signals is usually performed obtrusively, e.g., via physical sensors, or non-obtrusively, e.g., remote sensing based on computer vision.

#### 3.1.1. Physical Readings of Signals

Signals obtained by Electroencephalography (EEG), Electrocardiogram (ECG), Electromyography (EMG), and Galvanic Sking Response (GSR) are widely mentioned in the literature [58]. Reading of HR via ECG, for instance, have been shown to be usable in detecting emotional states [59], including stress [60] and boredom [9]. Mental stress or physical activity can influence the HR due to its connection to the ANS [61], which can be exploited for emotion detection. Facial activity is also mentioned as a source of information for emotion detection. The use of EMG to measure the electrical activity of facial muscles has been used to correlate emotions with activity of the zygomatic muscle [6], game events with activity of the corrugator [48], and the zygomatic and orbicularis oculi muscles [49]. Finally, non-invasive, watch-like wearable devices have also been demonstrated as sensors to collect physical signals for emotion classification [62]. Rincon et al. [63] demonstrate the use of such a wearable device that was custom-built to detect GSR, temperature, variations in blood flow, and movement. The device is jointly used with automated facial analysis and aims to detect the emotional state of individuals to collectively infer the emotional state of a group of users using a multi-agent approach. Emotion information of individuals using the wristband contributes to the calculations of an emotional state of the group they belong to.

Despite sensors allowing accurate measurement of signals, reports in the literature show that noise is still found in collected data due to sensor motion [64].

#### 3.1.2. Remote Readings of Signals

Contrary to the obtrusiveness of measuring signals using physical sensors, the extraction of signals using computer vision is presented as a viable, non-obtrusive alternative [65]. Remote photoplethysmography (rPPG) [66] is commonly used to estimate the HR of a person from the analysis of a video [67]. The principle of rPPG is the contact-less, camera-based detection and monitoring of the variations in light absorption that happen on the human skin in order to estimate cardiac activities [68]. The use of such remotely estimated HR measurements have been used in the context of emotion estimation, including the detection of cognitive stress [36], and inference of stress [3,24,25] and boredom [7]. The use of rPPG, however, is highly affected by the subject’s movement and facial activity, e.g., facial occlusion and talking, which is likely to happen during more natural interactions. Consequentially, evaluations of rPPG measurements are commonly performed in circumstances where subjects are instructed to remain still [18,69,70], or to perform artificial tasks intended to mimic natural behavior; examples of such tasks include arbitrary head movements [71], conduct mathematical operations mentally [36], and simplistic computer–human interactions [72,73]. Aiming to improve the accuracy of the HR estimations, rPPG-based techniques employ different approaches to collect and process the data acquired from a video. Initiatives include tracking of head movement [74], use of pre-defined skin-tone models [75,76], and signal estimation based on blind source separation (BSS) such as independent component analysis (ICA) [45]. Due to the statistical nature of ICA, the BSS-based technique by Poh et al. [45] is significantly resilient to subject’s motion and changes in illumination conditions, presenting better HR estimations compared to other rPPG techniques when subjects are not performing periodic motion [75], e.g., exercise.

Facial analysis performed manually or automatically is another source of information to extract user signals. When manual analysis is conducted, inspection commonly relies on the Facial Action Coding System (FACS) and its facial action units (AU) being used as a signals/features for quantitative emotion detection [77]. The frequency of manually annotated AUs has been reported as an indication of the emotional state of subjects, suggesting more AUs in stressful parts of a game compared to neutral parts [78]. Automated FACS-based analysis has been used to correlate engagement and frustration to facial expressions [79], as well as an estimator of emotional states in games [80]. The use of FACS-based analysis is contested; however, since it is a time-demanding task when performed manually and the decoding of facial expression has yielded different results due to cultural factors [81]. In that light, the automated analysis based on computer vision is presented as an alternative to detect facial features and classify them into facial expressions [82]. Some initiatives focus on detecting the six universal facial expressions [83], while others rely on distances, i.e., Euclidian distance, and angles of facial features to estimate emotional states [84,85,86,87,88,89]. The tracking of head movement and facial activity, e.g., talking and gestures, has also been reported as an important element in emotion detection, arguably because subjects feature a neutral face most of the time during the interaction with games [90]. Head movement is mentioned as another element for emotion detection, which can be used to detect gestures and facial expressions [91], or correlated with experience in gaming [92] or stressful situations [33].

### 3.2. Emotion Classification

Emotion classification based on psychophysiological signals commonly try to map those signals into emotional states defined by a model. One model mentioned in the literature is the basic emotions proposed by Ekman and Friesen [83]. Constructed from an experiment involving cultural differences, it suggests that particular facial muscular patterns and discrete emotions are universal. The six emotions mentioned in the theory are: happiness, surprise, sadness, fear, anger and disgust. A contrary definition is presented by Russell [93], who defined another model of emotions named Circumplex Model of Affect (CMA). Commonly referred to as Russell’s Arousal–Valence (AV) space, the model is contrary to strictly basic emotion models of affective state, where each emotion emerges from independent neural systems [94]. The model proposes a dimensional approach where all affective states arise from the activation of two fundamental neurophysiological systems: arousal (or alertness) and valence (a pleasure–displeasure continuum). In the field of games research, one of the most mentioned theories regarding emotions is the theory of flow. Flow was originally defined as a phenomenon in which a person experiences a subjective state characterized by an intense level of attention during the execution of an intrinsically motivated activity [95]. It has been used as the foundation for several concepts, including engagement and immersion [96], sense of presence [97] and applicability in game design [31,98,99].

#### 3.2.1. Approaches Based on Physical Contact and Sensors

The first multifactorial analysis approaches were based on obtrusive measurements of signals using physical sensors. Chanel et al. [100], for instance, demonstrate the use of multifactorial input analysis to measure emotions and involvement in a game context. In their study, participants play Tetris in different conditions of difficulty while a variety of sensors, including a respiration belt and an electroencephalogram (EEG) system, monitor them. Classification accuracy ranges from 48% to 55% depending on input signals and classifiers used to create the model.

Vandeput et al. [55] demonstrate the use of HR and HRV to detect mental and physical stress. Three demanding activities (a postural task, a mental task and a task that is a combination of both) are used and for almost all the HR measures obtained, the demanding activities can be distinguished from the rest period. The authors also point out that mental stress decreased high frequency components of the HRV interval, i.e., $HRV_{HF}$, while increasing low frequency ones, i.e., $HRV_{LF}$. A similar experiment conducted by Garde et al. [56] involved two tasks: one mental and physically demanding, i.e., digital version of the Stroop color word test [101], while the other was only physically demanding. The authors confirmed the findings of Vandeput et al. [55] by showing higher HR, increased $HRV_{LF}$ and decreased $HRV_{HF}$ during the mentally demanding task, compared to the rest period.

Grundlehner et al. [35] also use physical sensors to perform real-time continuous arousal monitoring. The authors record and use four signals from subjects to estimate arousal: ECG, respiration, skin conductance and skin temperature. The ECG is used to calculate HRV, which is then applied in the estimation. The data used for emotion-triggering is based on videos, sounds and a cognitive demanding game. A regression analysis is performed to identify the importance of the features in the estimation of arousal. $HRV_{LF}$ and $HRV_{HF}$ are not significant, compared to the other signals, e.g., skin conductance, while the standard deviation of HRV presents a significant weight. The arousal prediction matches the hypothesized arousal events marked by the authors in each of the emotion-triggering events. The results, however, were derived with controlled, pre-defined events that are expected to cause reactions, which might be related to arousal. More subtle or dynamic interactions, such as the ones obtained when a subject plays a digital game, might not be identified or detected by the approach proposed by the authors.

Bailenson et al. [30] use a combination of physiological signals (obtained from physical sensors) and facial expressions. Authors use a machine learning model to map the input signals to emotions (sadness or amusement). The training data used for the machine learning model are based on recordings of participants while they watched a video containing different emotion-triggering segments. Physiological signals, among them HR and skin conductance/temperature, are used in conjunction with video frames annotated by professional coders to create a predicting model. Results show a model better at categorizing emotions than measuring their intensities. In addition, the use of facial and physiological information combined is more efficient than using either one alone. Additionally, a person-specific model performs better than a group model, which suggests that person-tailored models might be more suitable for predictions than general-purpose ones.

#### 3.2.2. Approaches based on Remote, Non-Contact Analysis

Zhou et al. [102] propose a completely non-obtrusive and remote approach for user emotion identification based on a multifactorial analysis. A classifier is trained on a set of psychophysiological signals, among them HR and facial activity, i.e., expressions, blinking, pupil dilatation and head movement, in combination with information related to the user interaction, i.e., keyboard/mouse usage and sentiment analysis of the content being read. The accuracy rate the classifier achieves ranges from 56% to 89% depending on different emotional states being identified. However, the authors do not specify how much each input signals contributes to the emotional output. Additionally, it is not possible to infer whether such approach could be used outside the controlled environment created by the authors, since this would require a simulation of a social network filled with previously defined and known content in order to work.

McDuff et al. [36] also use a camera to remotely measure cognitive stress via HRV. Participants are recorded while resting and performing arithmetic tasks silently. A facial region is automatically detected and used to estimate the blood volume pulse (BVP) based on a PPG signal processed using Independent Component Analysis (ICA). A set of physiological parameters are then derived from the estimated BVP, including HR, HRV, and respiratory rate (RR). Such parameters are used to train a classifier to predict if a user is under cognitive stress. Results show a prediction accuracy of 85% for a model based on Support Vector Machine (SVM). Authors report that the HR was not significantly different during the periods of rest and cognitive stress. Additionally, RR and HRV are reported as the strongest predictors. McDuff et al. [24] perform further investigations but use different cognitive tasks (two cognitive demanding games). A person-independent machine learning model based on HR, HRV, $HRV_{LF}$, $HRV_{HF}$ (along with normalized and combined versions of these signals) and breathing rate is used to classify the stress level of the subjects. Authors report no statistical difference regarding heart and breathing rates in any case. The variations of $HRV_{LF}$ and $HRV_{HF}$, however, are significantly different during the cognitive tasks compared to the rest period; higher $HRV_{LF}$ and lower $HRV_{HF}$ power are found in both cognitive tasks compared to the rest period, which aligns with findings of previous work. The authors also point out that the stress predictions made by the model are consistent with the self-reported answers. The two participants with the highest self-reported stress level show the highest predicted stress level, while the two participants with the lowest self-reported stress level also present the lowest predictions.

Finally, Giannakakis et al. [33] present an approach for the detection of stress/anxiety on the basis of eye blink, mouth/head movements and HR estimations using rPPG. The participants are recorded while performing a set of tasks designed to trigger emotional responses, such as talking in a foreign language, remembering a sad incident, visualizing images/videos and playing a gamified cognitive test, i.e., Stroop test. Facial cues are obtained from an automatically detected region of interest (ROI). These cues are used to extract facial features connected to head movement, mouth and eyes. The extracted features, including HR information estimated using rPPG, are selected and ranked accordingly to maximize a machine learning classification step. Different classifiers are employed, which yield different classification accuracy rates. For each task performed by the subjects, classification accuracy ranges between 80% and 90% taking into account the most efficient classifier. It is noted by the authors that the observation of stressful images and the interaction with the Stroop test appear to be the most consistent across the classifiers employed.

## 4. Experimental Setup

We have conducted two distinct experiments, henceforth named Study 1 and 2, to systematically evaluate our method for remote detection of emotions. In Study 1, we aimed to test the feasibility of both our proposed method and the use of calibration games as emotion elicitation materials to train an emotion classifier. In Study 2, we aimed to evaluate the accuracy of our method in a more realistic scenario by applying it to estimate the emotional state of subjects during the interaction with a COTS game. Both experiments share common elements, such as the calibration games used for emotion elicitation. The following sections firstly describe such elements, followed by a detailed description of the unique elements of each study, including their setup and the profile of the subjects involved.

### 4.1. Calibration Games

**Figure 5:**
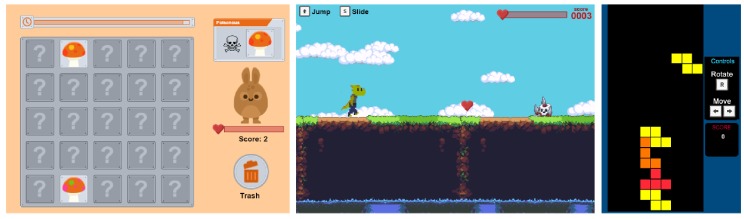
Screenshots of the three calibration games used in the experiments. From left to right: Mushroom, Platformer, and Tetris. Reproduced from Bevilacqua et al. [23].

Three games (source code available at: https://fernandobevilacqua.com/link/phd-experiment1) were used as stimuli elicitation materials in both experiments. They are illustrated in Figure 5. They were named *Mushroom*, *Platformer*, and *Tetris* due to their mechanics. The *Mushroom* game follows the puzzle genre and the main goal is to correctly sort poisonous from non-poisonous mushrooms. The distinction is made by the color pattern found on the upper part of the mushrooms. At each round, a set of mushrooms is presented to the player who has a certain time to correctly identify and sort them, dragging and dropping the good ones into a creature (the monster that feeds on the mushrooms) and the bad ones into the trash bin. At the upper part of the screen, the game shows an illustration of a poisonous mushroom that must be identified among the presented mushrooms for that round, along with a bar that shows the progression of time, i.e., time remaining to identify the mushrooms of the current round. At each round, a new illustration of a poisonous mushroom is presented along with a new set of mushrooms to be identified and sorted. The player does not have to remember previously presented poisonous mushrooms, just the currently presented poisonous one for the active round. As a consequence, a poisonous mushroom in a previous round could be a non-poisonous mushroom in future rounds, and vice versa. If the player performs a wrong action, e.g., drags a good mushroom into the trash or a bad one into the monster, a buzz sound is played, visual feedback informs about the mistake, and the health bar of the monster is decreased. Correct actions are rewarded with a pleasant sound, a visual feedback, and an increase in the health bar of the monster. If the player correctly identifies all mushrooms of the round before the time bar reaches the end, the player must wait until a new round is presented. If there are still mushrooms left to identify when the available time for the current round ends, each remaining mushroom is treated as a wrong action, i.e., a buzz sounds and visual feedback highlights all pieces that were not sorted. At the beginning of the game, rounds present few mushrooms and considerable time is available for sorting all pieces. As the subject plays the game and the time progresses, rounds present more mushrooms and less time is given to the sorting task. Eventually, mistakes will decrease the health bar of the monster towards zero, ending the game.

The *Platformer* game follows the endless runner platform genre and the main goal is to avoid obstacles while collecting hearts. The main character, i.e., a dinosaur, is displayed slightly to the left of the screen and the player is unable to move it horizontally. The player can make the character jump over or slide under obstacles. Platforms with different heights are presented to the player, moving from right to left; however, they never become disconnected, i.e., the player does not need to jump from one platform to another, just follow the provided slope up or down. When the main character collides with an obstacle, a sound related to hurting is played and the content of the health bar is decreased. If the character collects a heart, a pleasant sound is played and the content of the health bar increases. At the beginning of the game, the speed of the main character, i.e., how fast the platforms move towards the left, is significantly slow, few obstacles are presented, several hearts are available, and there are few variations regarding the heights of the platforms. As time progresses, the speed of the platforms increase, more obstacles, and different platform heights are presented, as well as less hearts being available. As the speed of the platforms and the number of obstacles increases, the player commits more mistakes, eventually leading the health bar of the main character to reach zero, which ends the game.

Finally, the *Tetris* game follows the genre of the original Tetris game. Differently from its original version, in our game, the player is not presented with a visual indication of the next piece to enter the screen. Additionally, the player is not able to use any key to speed up the movement of the pieces. They can only be moved to the right/left and rotated. At the beginning of the game, the speed the pieces fall is significantly low, so players must wait until pieces reach the bottom of the screen or fit into already established pieces. As time progresses, the speed the pieces fall increases every minute, so the player has less time to react to new pieces and plan their trajectory. Eventually, the pieces move too fast for the skill level of the player (which is different for each subject based on gaming experience, for instance), leading to mistakes and the pilling up of pieces. As in the original version of the Tetris game, when a piece reaches the upper part of the screen the game ends.

The three calibration games used the same seed for any calculation of randomness, i.e., random number generation. Consequentially, the same sequence of game elements was guaranteed to all subjects, e.g., same sequence of mushrooms in the Mushroom game, same pattern of platforms and obstacles in the Platformer, and same sequence of pieces in Tetris.

### 4.2. Equipment and Data Collection

**Figure 6:**
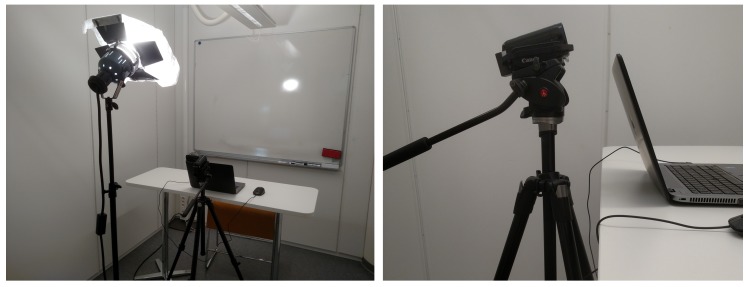
Experiment setup. On the left, an image illustrating the position of the equipment, including the angle of the external light source; on the right, an image highlighting the position and angle of the video camera.

In both experiments, subjects were seated alone in a room in front a computer, while being recorded by a camera and measured by a heart rate sensor. The camera was attached to a tripod placed in front of the subjects at a distance of approximately 0.6 m; the camera was tilted slightly up. A spotlight, tilted 45${}^{\circ }$ up, placed at a distance of 1.6 m from the subject and 45 cm higher than the camera level, was used for illumination; no other light source was active during the experiment. Figure 6 illustrates the setup.

During the whole experiment, subjects were recorded using a Canon Legria HF R606 video camera (Canon Inc., Tokyo, Japan). All videos were recorded in color with 24-bit RGB, i.e., three channels of color with eight bits each, and a frame rate of 50 p. Video files presented a resolution of 1920 × 1080 pixels and used the AVCHD-HD format (encoded using MPEG-4 AVC). At the same time, the HR of subjects was measured with a frequency of 1 Hz by a TomTom Runner Cardio watch (TomTom International BV, Amsterdam, The Netherlands). The watch was placed on the left arm like a regular wrist watch at a distance of about 7 cm from the wrist. The use of such device was unobtrusive, therefore subjects could still freely use both hands to play the games.

After playing each calibration game, subjects answered a questionnaire to provide self-reported emotional states related to stress and boredom. Questions were presented as a 5-point Likert scale contextualized regarding the beginning and the end parts of the games, e.g., how stressful was this game at the beginning (1: not stressful at all, 5: extremely stressful). After playing all games, subjects also answered a questionnaire about demographics and gaming experience. The questions were based on the Video Game Experience Questionnaire [103] and the Survey of Spatial Representation and Activities [104].

### 4.3. Participants

In both experiments, subjects were recruited through advertisement material spread on social media channels and in the campus of the University of Skövde. Subjects were students, staff members, and inhabitants of the community/city. All participants gave their informed and written consent to participate in the experiments, which were approved by the research ethics adviser at the University of Skövde, Sweden and conducted in accordance with the ethical recommendations of the Swedish Research Council [105]. There were, for Study 1 and 2 respectively, n=20 subjects (50% female) aged 22 to 59 years old (mean 35.4 ± 10.79) and n=62 subjects (38.7% female) aged 19 to 66 years old (mean 27.2 ± 7.2). Participants of this experiment, i.e., Study 2, and those of Study 1 are different; there is no overlap of subjects in both experiments. The population of subjects in both experiments presented a diversity of gender and gaming experience, as shown in Table 2 and Table 3, which present self-reported levels of skill at playing video games and the number of hours per week subjects played any type of video game over the last year for subjects of Study 1 and 2, respectively.

The diversity in gaming experience, gender and age of the subjects in both of our experiments provides heterogeneous data that allow a more realistic and broad analysis of our proposed method. It is important to highlight that, in a model trained on a group basis, i.e., data from several subjects are used to train a model that is evaluated on other subjects, the wide range of ages and gaming experiences of our population of subjects could create outliers in the statistical examination of the training and testing phases. This is not the case since our method uses a user-tailored approach, i.e., the emotion classifier is trained and evaluated on the data of a single subject, not a group. Consequentially, all statistical analysis conducted in the training and testing phases is based on each subject, which means that a given subject is evaluated with a model trained on the data from that same given subject. In summary, the answers given by a particular subject are the baseline for the analysis of that subject.

### 4.4. Configuration of Study 1

#### Materials and Procedures

Each participant was recorded for approximately 25 min, during which they played three games, i.e., Mushroom, Platformer and Tetris. Subjects played those three games in a randomized order. The first two games were followed by a 138 s’ rest period, during which subjects listened to calm classical music. Participants received instructions from a researcher informing them that they should play three games, answer a questionnaire after each game and rest. In addition, they were told that their gaming performance was not being analyzed, that they should not give up in the middle of the games and that they should remain seated during the whole process. As previously mentioned, subjects answered a questionnaire after playing each game.

### 4.5. Configuration of Study 2

#### 4.5.1. Materials and Procedures

**Figure 7:**
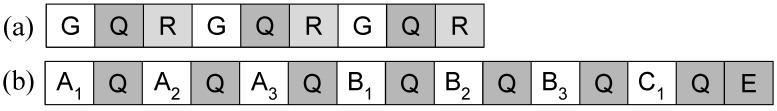
Two (uninterrupted) parts of the experiment. (**a**) calibration part; (**b**) testing part. G: calibration game, Q: questionnaire about game/level, R: resting, ABC: levels of Infinite Mario, E: demographic questionnaire.

Participants were recorded for an average of 45 min during two (uninterrupted) parts of the experiment, i.e., the calibration and testing phase, as illustrated by Figure 7. In the calibration part, aimed at gathering data for training a user-tailored model, subjects played three calibration games (described in Section 4.1). Subjects played those calibration games in a randomized order. Similarly to Study 1, after playing each game, subjects answered a questionnaire and rested for a period of 138 s. In the testing part, aimed at gathering data to test the accuracy of the user-tailored model, subjects played seven levels of an evaluation game, i.e., Infinite Mario (described in Section 4.5.2). Subjects answered a questionnaire after each level of Infinite Mario to provide a self-reported assessment of experienced stress and boredom. The questionnaire had two questions, one concerning stress and the other concerning boredom; both used a 5-point Likert scale to assess the level of stress/boredom players experienced during the level played, e.g., 1 being not bored at all, 5 being extremely bored.

Regarding the levels of Infinite Mario in the testing part, they were organized in three batches: batches A, B and C containing 3, 3 and 1 level each, respectively. The levels in batches A and B were designed to present an increase in difficulty within the batch; therefore, levels A${}_{1}$ and B${}_{1}$ were expected to be less difficult/challenging than levels A${}_{3}$ and B${}_{3}$, for instance. Similarly, the levels in batch B were designed to be more challenging than the levels in batch A, also following an increase in difficulty. Consequently, levels B${}_{i}$ are expected to be slightly more difficult/challenging than levels A${}_{i}$. This pattern intended to mimic the balance curve of a commercial game, where levels, and game parts, commonly tend to increase their difficulty as the game story progresses. In order to ensure that the subjects would experience some level of boredom during the testing phase, which is required for the evaluation of the proposed method, levels B${}_{1}$ and C${}_{1}$ were designed using a particular set of changes, including the use of Mario’s auto-scrolling camera mechanics. In such a configuration, the player has no control of the speed of the level. After each level was played, the subjects were required to answer a questionnaire about how boring/stressful the game level had been. The order in which the levels were played was not randomized among the subjects during the testing phase of the experiment. As a consequence, all the subjects played the evaluation game in the same order: levels A${}_{1}$ to A${}_{3}$, then by levels B${}_{1}$ to B${}_{3}$, finally level C${}_{1}$.

After subjects finished playing the last level in the testing part, i.e., level C${}_{1}$, they answered a final questionnaire about their age and gaming experience/profile. Before starting the experiment, participants received the following instructions from a researcher: they should play a few games, answer questionnaires after each game and rest when instructed; they were told that their gaming performance was not being evaluated that they should not give up in the middle of the games that a time limit exists for the levels to prevent them from playing too long, and that they should remain seated during the whole process.

#### 4.5.2. Evaluation Game: Infinite Mario

The game used in the evaluation phase of the experiment is a modified version of Markus Persson’s Infinite Mario, a public domain clone of Nintendo’s platform game *Super Mario Bros* (the version of the game used in the experiment is an HTML5, web-based version built by Robert Kleffner, available at: https://github.com/robertkleffner/mariohtml5; Robert ported to HTML5 the original Java version created by Markus Persson; source code of both versions, Robert’s and Markus’, are in the public domain; the source code of the final version used in this experiment is available at: https://fernandobevilacqua.com/link/phd-experiment2). In the case of this experiment, the game is played with a keyboard in a web browser. Infinite Mario has been widely mentioned in the literature, including studies involving the modeling of player experience [92,106,107] and detection of affective states [108].

The gameplay in Super Mario, consequentially in Infinite Mario as well, consists of controlling the main character, Mario, along the level. Mario can move left or right, jump, run, duck, and throw fireballs (if the power-up *Flower* has been collected). The objective of the game is to complete each level, which is accomplished by traversing it from left to right until the “end of level” checkpoint is reached. Mario can be in three different states: small, big, and power-up. If Mario is small, any interaction with enemies that is different from landing on top of them after a jump results in Mario getting killed immediately. If Mario is big, the same “wrong” interaction with enemies causes Mario to be hurt and transform into the small state. If Mario is in the power-up state, the “wrong" interaction with enemies causes Mario to be hurt and transform into the big state. Consequently, keeping Mario in the big or power-up state is a strategic advantage that prevents Mario from being killed, which is likely to calm players, i.e., relaxed emotional state. On the other hand, keeping Mario in the small state is less beneficial, since mistakes are fatal, thus likely causing players to feel anxious/stressed in such conditions. Along the level, Mario might encounter enemies, which can be killed or ignored. Mario can kill enemies by jumping and landing on top of them, which is rewarded with score points. Some enemies, e.g., Koopa Troopa (a sort of turtle), leave a shell behind when killed by Mario. The shell can be picked up by Mario and carried around, serving as a weapon when released. The levels might also contain terrain obstacles of varying sizes, e.g., gaps that must be jumped over. If Mario falls into a gap, he dies immediately. Mario can also find collectable items, i.e., coins and power-ups, or interactable items, e.g., blocks. Mario collects items by touching them, i.e., a collected coin results in score points. Collectable items might be visible in the level or hidden inside interactable items, e.g., blocks. Mario interacts with blocks by bumping into them from below, e.g., jumping and hitting Mario’s head on the bottom of a block destroys it. A destroyed block might give a collectable item as a reward, e.g., coin, *Mushroom* (Mario transitions to big state) or *Flower* (Mario transitions to power-up state).

During gameplay, information about Mario, the score and the current level is displayed at the top of the screen. This information includes the number of lives Mario has left to complete the level, the level score, number of coins collected (collecting 100 coins results in an extra life), name of the current level, and the amount of time available to complete the level (constantly ticking down). When the time remaining to complete the level reaches the 60 s mark, a hurry up sound is played, then the background music starts to play in a faster tempo. Unless informed otherwise, all the levels of Infinite Mario in the experiment start with three lives and 200 s of available time. Every time Mario dies, the time remaining to complete the level is reset to its initial value, e.g., 200 s.

Originally, Infinite Mario procedurally generates all its gameplay content, e.g., level design and position of items/enemies. This behavior was not desired for the experiment, since all the subjects should experience the same Mario levels. Additionally, subjects should feel stressed and bored in the game at some point, so that the proposed emotion detection method can be properly evaluated when such moments are detected. As a consequence, Infinite Mario was adapted and tweaked, thus made to fit as an ideal evaluation game in the experiment. The procedural content generation was constrained by a seed and a set of parameters was introduced to control the creation of the content, e.g., length of the level, amount and width of terrain obstacles, such as gaps and platforms, availability of coins and power-ups, among others. It ensured that all the subjects experienced exactly the same levels.

**Figure 8:**
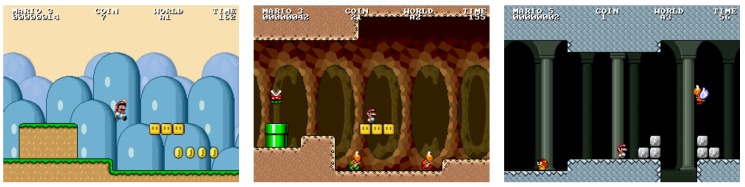
Screenshots from Infinite Mario. From left to right, level types *Overground*, *Underground* and *Castle*, respectively.

Previous works using Infinite Mario [106,107] have shown a correlation between anxiety and (1) difficulty of jumping, e.g., overcoming obstacles, and (2) gap width. There is also a correlation between boredom and the width of gaps, i.e., the wider the gap, the less boring the level. Based on those findings and the guidance provided by game design experts, the Mario levels used in the experiment were adjusted according to the description presented in Table 4. Column *Level* refers to the level name/number. Column *Type* refers to the overall visual representation of the level. Possible types are *Overground* (open sky and green landscape), *Underground* (closed ceiling, dirt-like environment), and *Castle* (closed ceiling with bricks resembling the interior of a castle). Each level type features different background music and visual elements, as illustrated in Figure 8. Column *Emotion* refers to the expected emotional state most subjects will experience. Finally, column *Adjustments* refer to the constraints used to generate the levels content.

Level $A_{1}$ is an introduction to the game to familiarize the subjects with the mechanics, e.g., move, jump, collect items. Levels $A_{2}$ and $B_{2}$ were designed to be regular Mario levels with a compelling and enjoyable challenge scale. Levels $A_{3}$ and $B_{3}$ were designed to be more stressful by including more enemies and several gaps which were wider than usual. The absence of power-ups, the number of challenges, i.e., enemies and wide gaps, and the fact that Mario is continuously in the small state should force the subjects to better time actions, e.g., jump, and constantly pay attention to the surroundings. These levels also use the *Castle* type, which is usually associated with “boss levels” in Super Mario (commonly more challenging). Finally, levels $A_{3}$ and $B_{3}$ have an available time of 80 s to be finished, a considerably lower value compared to 200 s in other levels. As a consequence, after 20 s of gameplay, the hurry up sound is played and the background music starts to play faster, a configuration that is likely to cause an emotional state of stress. In contrast, levels $B_{1}$ and $C_{1}$ were designed to be more boring. These levels include an auto-scrolling camera mechanic, which enables the camera to automatically traverse the level independently of Mario’s movements. The speed of the auto-scrolling camera was adjusted to be at constant, but slow pace. Additionally, the reduced number of interactable/collectable items, the existence of only a few terrain obstacles, as well as the absence of gaps, power-ups and enemies are likely to cause an emotional state of boredom. Furthermore, levels $B_{1}$ and $C_{1}$ are very similar visually, which might cause subjects to perceive level $C_{1}$ as a repetition of level $B_{1}$. In that case, subjects might perceive level $C_{1}$ as even more boring, since the level topology is already known and the player is unable to move the camera at a faster pace.

As previously mentioned, the levels were adjusted and play-tested by game design experts. It ensured that the content of all levels and the constraints/modifications applied to them did not affect the subject’s perception of playing a clone of a Mario. For instance, the order in which the levels were played, i.e., repeating the pattern of an overground, then an underground, and finally a castle level, was kept as an important element. It should mimic the expected world progression of the original Mario game, where the final level of a particular world is usually a castle level with a boss. Finally, particular attention was invested to make Infinite Mario levels difficulty as different as possible from the linear difficulty progression present in the three calibration games. The aim was to make Infinite Mario as similar to Super Mario as possible respecting the content constraints mentioned previously.

## 5. Analysis and Procedures

The following sections describe how the data gathered in both studies, including the video recordings and the self-reported emotional states, were processed and analyzed to evaluate our proposed method.

### 5.1. Study 1

The aim of Study 1 is to evaluate the feasibility of using calibration games as emotion elicitation materials to train a neural network able to classify emotional states of stress and boredom. In this study, subjects played three calibration games, which were used both to train and evaluate an emotion classifier based on our proposed method. Additionally, Study 1 aims to assess how different features influence the classification process, for instance, if the combined use of HR and FA features is better than the use of FA only. The assessment also included the use of HR data from a physical sensor as a classification feature, so the impact of using a physical sensor instead of rPPG could be checked regarding classification accuracy.

#### 5.1.1. Data Pre-Processing

**Figure 9:**
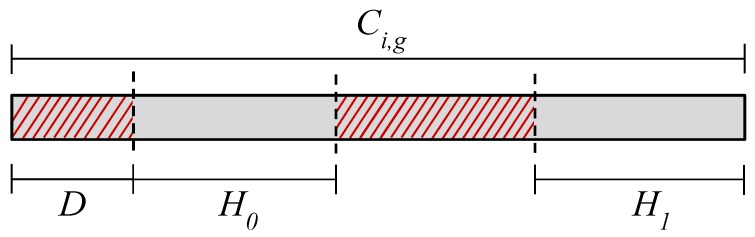
Extraction of video segments $H_{0}$ and $H_{1}$ containing boring and stressful interactions, respectively, in the calibration games. Initial *D* seconds of any video $C_{i,g}$ are ignored and the remainder is divided into three segments, from which the first and the last ones are selected. Stripes highlight discarded video segments.

The pre-processing of video recordings involved the extraction of the parts containing the interaction with the games and the discarding of noisy frames. The process is illustrated in Figure 9. Firstly, the periods in which the subjects played each of the available games were extracted from the video recordings. This resulted in three videos per subject, denoted as $C_{s,i}$ where *s* is the *s*-th subject and i∈{1,2,3} represents the game. Then, the initial D=45 s of any given video $C_{s,i}$ were ignored because we assumed it might not be ideal for emotion analysis. Firstly, during that period, subjects are less likely to be in their usual neutral emotional state. They are more likely to be stimulated by the excitement of the initial contact with a game soon to be played, which interferes with any feelings of boredom. Secondly, subjects need a basic understanding of and experimentation with the game, in order to assess whether it is boring or not. As per our understanding, it is less likely for such conjecture to be fulfilled during the initial 45 s of gameplay than it is afterwards. After the removal of *D* seconds, the remainder of each video $C_{s,i}$ was divided into three segments, from which the first and the last were selected as $H_{0}$ and $H_{1}$, respectively. Since calibration games were designed to be boring at the beginning and stressful at the end, segments $H_{0}$ and $H_{1}$ are more likely to represent the moments subjects perceived the games as being boring and stressful, respectively.

The pre-processing of the recordings resulted in six video segments per subjects: three segments $H_{0}$ (one per game) and three segments $H_{1}$ (one per game). A given game *i* contains n=20 pairs of $H_{0}$ and $H_{1}$ video segments (20 segments $H_{0}$, one per subject, and 20 segments $H_{1}$, one per subject). Regarding all the subjects and games, there are n=60 pairs of $H_{0}$ and $H_{1}$ video segments (3 games × 20 subjects, resulting in 60 segments $H_{0}$ and 60 segments $H_{1}$). Subject 9 had problems playing the Platformer game; therefore, all segments $H_{0}$ and $H_{1}$ from that subject were discarded. Consequentially, there are n=57 pairs of $H_{0}$ and $H_{1}$ video segments in total, after the pre-processing.

The emotion elicitation design of the calibration games, where $H_{0}$ and $H_{1}$ represent boring and stressful interactions, respectively, is used to label samples to train and test the model. Samples from the $H_{0}$ part were labeled as boredom and samples from the $H_{1}$ part were labeled as stress. This process accounts for the informed levels of boredom and stress of the subject, which aimed to ensure a correct labeling of the samples, based on video segments that more likely, accurately reflect the emotional state self-reported by the subjects.

#### 5.1.2. Features Extraction and Calculation

Features used in the classification model were extracted remotely via the analysis of the video segments of each subject. In total, nine features, denoted $F_{1}$ to $F_{9}$, were used in the process. Detailed information regarding how features $F_{1}$ to $F_{8}$ were extracted, calculated, and aggregated, as well as the reasoning for its use is presented in Section 2.2. Feature $F_{9}$, similarly to feature $F_{8}$, represents HR activity, however it is calculated on the basis of the HR measurements performed by a physical sensor, i.e., watch, used by subjects. It is important to highlight that feature $F_{9}$ is not part of our proposed method for remote detection of emotions. It has been used as a classification feature in study 1 to evaluate to what extent a HR measurement that is not based on a rPPG estimation, i.e., a physical sensor in this case, effects the emotion classification.

#### 5.1.3. Training and Evaluation of an Emotion Classifier

**Figure 10:**
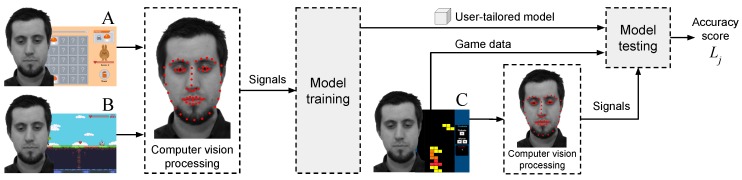
Iteration of a 3-fold *Leave-One-Session-Out Cross-Validation* performed on the gaming session of a given subject with three games, i.e., A, B and C. Data of two calibration games, e.g., A and B, are used to train the machine learning model, while data of the third calibration game, e.g., C, are used to evaluate the model.

The classification procedure uses the previously mentioned features $F_{1}$ to $F_{9}$ and a neural network trained to identify two emotional states: boredom and stress. Both the training and evaluation of the neural network are performed in a user tailored fashion: data from a given subject $S_{i}$ is used to train and evaluate the emotion classification of that given subject $S_{i}$. Figure 10 illustrates the process.

Leave-One-Session-Out Cross-Validation (LOSOCV) is used to evaluate each trained user-tailored model, as illustrated in Figure 10. In LOSOCV, the data from one session instance are left out and a model is constructed on the data from all other session instances. In the present study, a given subject $S_{i}$ played three calibration games, e.g., A, B and C, thus, the data from one calibration game are left out and a model is trained on the data of the other two calibration games for that subject $S_{i}$. This is repeated for all three calibration games of that subject $S_{i}$. Consequentially, the use of LOSOCV will produce three models per subject, resulting in three measurements of classification accuracy per subject, denoted $L_{j}$, where j∈{1,2,3} represent each evaluated model. The final classification accuracy for given subject $S_{i}$, named $A_{i}$, is calculated as the mean of $L_{j}$ values obtained from the iterations in the LOSOCV. In other words, each subject contributes a single classification accuracy value $A_{i}$, which is calculated on the basis of the mean classification accuracy of the subject’s three models in the LOSOCV iterations.

In the training process of each model, which is performed three times per user, the hyper- parameters of each neural network, e.g., number of neurons, are optimized using random search [109]. A 10-fold cross validation method repeated three times is applied, which divides the dataset into 10-subsets, each of which is left out while the model is trained on all the others. The process is repeated three times and the final metric for the model is the mean from the number of repeats. The area under the ROC curve (AUC) is used as a metric to select the best model.

As previously mentioned, the calibration games were perceived as boring at the beginning and stressful at the end. As a consequence, it is assumed that the subject’s emotional state in $H_{0}$ and $H_{1}$ is boredom and stress, respectively. Based on this assumption, training and evaluation data obtained from the video segments in $H_{0}$ and $H_{1}$ were labeled as boredom and stress, respectively.

#### 5.1.4. Analysis

In order to test the effectiveness of the neural network in classifying samples as either boredom or stress, all of the trained neural networks were evaluated in conjunction. As described in the previous section, each subject’s model was evaluated using LOSOCV, which produced a classification accuracy $A_{i}$ for any given subject $S_{i}$. The minimum, maximum and mean value of $A_{i}$ was calculated as a metric for accuracy. In order to better contextualize the classification results, the same process was also applied to the other three metrics obtained during the LOSOCV evaluation: Precision, Recall and F1 score (F1 score should not be confused with $F_{1}$, the mouth outer facial feature used in the model). Precision accounts for the correctly predicted positive observations of the total predicted positive observations, e.g., of all the samples classified as stress, how many were indeed labeled as stress. Recall accounts for the correctly predicted positive observations of all the available observations in a class, e.g., of all the available samples labeled as boredom (or stress), how many were actually classified as such. Finally, the F1 score is the weighted average of Precision and Recall.

In order to better understand the contribution of each feature for the classification process, the training/evaluation process mentioned previously was also performed using different feature sets. Each of these different feature sets was evaluated in an independent test, denoted $T_{i}$. Table 5 shows tests $T_{i}$ and the corresponding feature sets used in the process.

Tests MULTI_R and MULTI_G use a multifactorial set of features for their neural network, where facial and HR information is used in combination. The difference between MULTI_R and MULTI_G is that the former uses rPPG estimated HR, while the latter uses the HR obtained from the physical sensor. Test FACE uses a set of features based solely on facial information. Finally, tests HR_R and HR_G use only HR information as a feature. Similar to MULTI_R and MULTI_G, tests HR_R and HR_G use HR readings from rPPG estimations and a physical sensor, respectively.

Since the calibration games used in the experiment induce emotional states of boredom and stress, then this difference should enable a trained neural network to properly classify evaluation samples as either boredom or stress. Additionally, the use of a multifactorial approach, where facial analysis and HR information are used in combination instead of either one alone, is expected to produce better classification results [8]. Based on those expectations, the following hypotheses state:

- $u_{1}$: a user-tailored neural network using a multifactorial feature set, i.e., facial and HR features, performs with greater accuracy than a user-tailored neural network using facial features only;
- $u_{2}$: a user-tailored neural network using a multifactorial feature set, i.e., facial and HR features, performs with greater accuracy than a user-tailored neural network using HR features only.

Hypotheses $u_{1}$ and $u_{2}$ were tested by performing a Wilcoxon Signed Ranks test on all $J_{i}$ values of the two competing tests $T_{i}$. As previously mentioned, the use of LOSOCV produces 57 accuracy measurements $J_{i}$ per test $T_{i}$.

### 5.2. Study 2

The aim of Study 2 is to validate the proposed method for remote detection of emotions in a larger scale compared to Study 1. In Study 1, subjects interacted with three calibration games; in the training (or calibration) phase, two of those calibration games were used to train the emotion classifier, while the third calibration game was used in the testing phase. In Study 2, our method is evaluated using the same two phases, i.e., training (or calibration) and testing; however, the three previously described calibration games are used to train the emotion classifier, while a fourth game, i.e., Infinite Mario, is used for evaluation. In summary, during the training phase, the model is trained by applying a user-tailored approach, i.e., data from subject $S_{a}$ playing three calibration games (Mushroom, Platformer and Tetris) are used to create model $N_{a}$. The result of the training phase is a user-tailored model, i.e., model $N_{a}$, a trained neural network for use on subject $S_{a}$. The testing phase occurs in a game session involving subject $S_{a}$ playing the non-calibration game Infinite Mario. During the testing phase, the signals of subject $S_{a}$ are remotely acquired and fed into the previously trained model $N_{a}$, which outputs the estimated emotional state of subject $S_{a}$ for that particular testing game. In summary, the aim of this experiment is to answer the following research question: how accurate is an emotion detection approach that uses remotely acquired signals, i.e., heart rate and facial actions, as input of a machine learning model, i.e., neural network that is trained on a user-tailored basis (one subject produces one model) using calibration games as emotion elicitation? The overall goal of this experiment is to analyze the emotion classification accuracy during the testing phase, which is more similar to a real gaming session involving a commercial game, for instance. The following sections present a detailed explanation of the experiment, including how data was processed and analyzed.

#### 5.2.1. Data Preprocessing

Before any analysis was conducted, the video recordings of the experiment were pre-processed to allow the extraction of training and validation data. The process involved the extraction of the parts containing the interaction with games and levels, as well as the discarding of noisy frames. The pre-process procedure is notably similar to the one described in Section 5.1.1. The periods during which subjects played the available games and levels were extracted from the video recordings. The pre-processing of the calibration phase, illustrated in Figure 9, resulted in three videos per subject, denoted $C_{i,g}$ where *i* is the *i*-th subject and g∈{1,2,3} represents a calibration game. The initial D=45 s of any given video $C_{s,i}$ were removed since they were deemed noisy regarding emotion information. The remainder of the video was then divided into three segments, from which the first and the last were selected as $H_{0}$ and $H_{1}$, respectively. The middle part was discarded because its emotional state is unknown. Segments $H_{0}$ and $H_{1}$ represent the boring and stressful parts of the calibration games, respectively. The pre-processing of the testing phase resulted in seven segments per subject, denoted $M_{i,m}$ where *i* is the *i*-th subject and m∈{1,2,…,7} represents a level of Infinite Mario. Video segment $M_{1,4}$, for instance, represents the recording of subject 1 playing level $B_{1}$. No parts were discarded from video segments collected in the testing phase.

The pre-processing of all the recordings resulted in 13 video segments per subject: three segments $H_{0}$ (one per calibration game), three segments $H_{1}$ (one per calibration game), and seven segments *M* (one per level of Infinite Mario). Considering all the subjects, the pre-processing of the calibration phase resulted in n=186 pairs of $H_{0}$ and $H_{1}$ video segments (3 calibration games × 62 subjects, resulting in 186 segments $H_{0}$ and 186 segments $H_{1}$). The testing phase resulted in n=434 video segments *M* (seven levels of Infinite Mario × 62 subjects).

#### 5.2.2. Features Extraction

Features used in the classification model were extracted remotely via the analysis of the video segments of each subject. In total, eight features, denoted $F_{1}$ to $F_{8}$, were used in the process. Detailed information regarding how each feature was extracted, calculated, and aggregated, as well as the reasoning for its use, is presented in Section 2.2.

#### 5.2.3. Training of the Emotion Classifier

**Figure 11:**
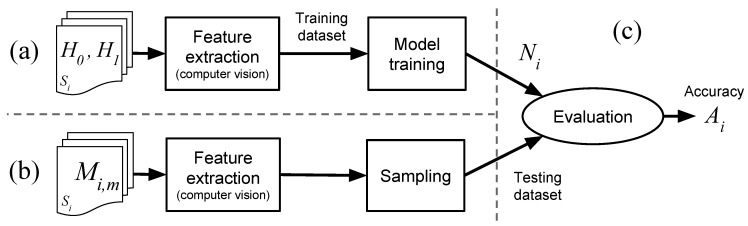
Training and evaluation of a user-tailored emotion classifier. (**a**) training of the emotion classifier; (**b**) construction of a testing dataset; (**c**) evaluation of the emotion classifier.

The classification procedure uses the previously mentioned feature set and a neural network to identify two emotional states: boredom and stress. Both the training and evaluation of the neural network were performed on a user-tailored basis: data from the calibration games of a given subject $S_{a}$ were used to train a model for that subject, i.e., model $N_{a}$, which is then used to classify the emotional state of that given subject $S_{a}$ on levels of Infinite Mario. Figure 11 illustrates the process.

In the training process of each user-tailored model, illustrated in Figure 11a, features are extracted from the video segments $H_{0}$ and $H_{1}$ (calibration data) of a given subject. This information is used to create a training dataset for that given subject. Since the calibration games induce boredom at the beginning and stress at the end of its interaction, as detailed in Section 4.1, it is assumed that a subject’s emotional state in $H_{0}$ and $H_{1}$ is boredom and stress, respectively. Based on this assumption, training data obtained from video segments in $H_{0}$ and $H_{1}$ were labeled as boredom and stress, respectively.

The training dataset was then used to train the user-tailored model, i.e., neural network. The hyper-parameters of the subject’s model, e.g., number of neurons, were optimized using random search [109]. A 10-fold cross-validation method repeated three times was applied, so the training data was split into 10-subsets and each of those subset is left out while the model was trained on all the others. The process is repeated three times and the final metric for the model is the mean from the number of repeats. The area under the ROC curve (AUC) was used as a metric to select the best model.

The result of the training process is a trained neural network, i.e., $N_{i}$ where *i* is the *i*-th subject. The model is said to be user-tailored because it was trained using only data from a given subject, e.g., subject $S_{a}$ produces model $N_{a}$.

#### 5.2.4. Construction of a Testing Dataset

The majority of the works in the literature validate an emotion classifier by applying it to a share of the samples not used for training. Generally, all available data samples are split into two sets, e.g., one with 80% and one with 20% of all the samples, which are then used for training and testing/validation, respectively. In such a configuration, all data used in the process come from the same source, the only difference is how the data are distributed in the different sets. In contrast to that approach, the evaluation of the emotion classifier proposed in this experiment was validated using a completely different and independent dataset.

As mentioned in the previous section, data extracted from the calibration games, i.e., video segments $H_{0}$ and $H_{1}$ of a given subject $S_{a}$, are used to train a model $N_{a}$. On the other hand, data extracted from the Infinite Mario game, i.e., video segments $M_{a}$ of subject $S_{a}$, are sampled to produce a testing dataset. It is important to highlight how unique and challenging such a configuration is, since the user-tailored model is trained on one kind of dataset (calibration games) and tested/validated on another (evaluation game). Each dataset is derived from different and independent sources. Despite this configuration, the game used for evaluation, i.e., Infinite Mario, still shares common characteristics with the calibration games, such as the 2D and casual mechanic.

The feature extraction procedure described previously uses a moving window of 15 s with a step of one second. When it is applied to the video segments $M_{i}$, a new value for each feature is extracted per second. It has been reported in the literature that HR-based emotion estimation is possible every 10 s [110]; however, reports also show changes in the inter-beat interval of HR within four seconds after in-game events [49]. Regarding facial actions, they might change faster than the HR; therefore, it is reasonable to believe they could significantly change within a time span of 10 s. Based on that information and insights gained from Study 1 regarding changes of signals, a sampling of five seconds was selected to collect feature values for the testing dataset of a given subject from video segments $M_{i,m}$. A sampling of five seconds was expected to cover changes both in HR and facial actions as often as possible, without risking the collection of samples that are not independent.

The testing dataset of a given subject $S_{a}$ contains samples (acquired every five seconds) from all the levels of that given subject $S_{a}$ selected for evaluation. The self-reported emotional state provided by each subject in the selected levels is used as ground truth to test the accuracy of the model. The levels of Infinite Mario used for evaluation are selected according to the following procedure. Assuming that $rstress_{i,j}$ and $rboredom_{i,j}$ represent the self-reported levels of stress and boredom of subject $S_{i}$ in a Mario level *j*, respectively, a stress score $stress_{i,j}$ and a boredom score $boredom_{i,j}$ are calculated as:

(1) $$stress_{i,j}=rstress_{i,j}-rboredom_{i,j},$$

(2) $$boredom_{i,j}=rboredom_{i,j}-rstress_{i,j}.$$

Two Mario levels, i.e., video segments $M_{i,m}$, of a given subject *i* with the highest values for $stress_{i}$ are selected and used to sample the stress entries. Similarly, the two levels with the highest values for $boredom_{i}$ are selected and used to sample the boredom entries. In order to avoid sampling levels whose self-reported emotional state is inconclusive, e.g., stress and boredom levels are equal, the levels already selected for sampling, whose values of $stress_{i}$ or $boredom_{i}$ are not greater than or equal to 1 are excluded from the sampling process.

#### 5.2.5. Evaluation of the Emotion Classifier

Similar to the training process, the evaluation process happens on a user basis, as illustrated by Figure 11c. After the user-tailored model $N_{a}$ of a given subject $S_{a}$ has been trained, it is applied to the testing dataset of that subject. The testing dataset of a given subject $S_{a}$ contains all the samples (acquired every five seconds) from all the levels of that given subject $S_{a}$ selected for evaluation, as described in Section 5.2.4.

The evaluation of the proposed emotion classifier is based on classification accuracy. As mentioned previously, each user-tailored model $N_{i}$ is applied to a testing dataset sampled for that particular subject. Consequentially, each subject $S_{i}$ produces one single accuracy metric, named $A_{i}$. The overall classification accuracy of the proposed method is calculated on the basis of the mean of all $A_{i}$ values.

#### 5.2.6. Analysis

The aim of the experiment is to validate the proposed emotion detection approach, i.e., use of remotely acquired signals and a user-tailored model (trained on data from calibration games) to detect emotional states of stress and boredom. The validation of the approach will be tested in terms of classification accuracy. Thus, the following hypothesis states:

- $u_{3}$: a user-tailored model, i.e., neural network, trained on data samples from three calibration games of a given subject $S_{a}$, i.e., Mushroom, Platformer and Tetris, is able to classify the emotional state of samples extracted from an evaluation game, i.e., Infinite Mario, played by that same subject $S_{a}$ with a mean accuracy greater than the chance-level rate.

The chance level is thereby the accuracy achieved assuming it is equally likely for a data sample to fall in any of the existing classes [111]. For a balanced two-class problem, the chance-level classification accuracy equals 50%. However, a chance-level accuracy rate of 50% assumes that a classifier performs random guessing on data-sets of infinite size. As a consequence, random guessing approximates chance-level accuracies, if the testing data set is large enough. If the testing data set is small, random classification can deliver accuracies that significantly deviate from chance level [112]. In order to account for that, a minimal correct classification rate for accuracy has been calculated using the binomial cumulative distribution to assert statistical significance with a confidence level of 95% (p<0.05) as a function of sample size *n*, i.e., size of validation dataset, and number of classes, i.e., boredom and stress [112]. Since the subjects had different gaming skills, the time spent playing the levels of Infinite Mario was likely to differ. This variance produced validation datasets of different sizes among the subjects. An analysis of all the validation datasets shows a mean size of 64.4 samples with 32.1 and 32.3 as the mean number of stress and boredom samples in each set, respectively. Therefore, it was assumed that the proposed method was to be validated as a balanced two-class problem with a sample size of n=64 (on average) used for the evaluation of each classification. Based on these numbers, a mean classification accuracy rate of 60% was found to be the minimal rate to assert classification better than chance-level. Consequentially, the null hypothesis associated with $u_{3}$ is: a user-tailored model trained on data samples from three calibration games of a given subject $S_{a}$ is not able to classify the emotional state of samples extracted from an evaluation game played by that same subject $S_{a}$ with a mean accuracy greater than chance-level rate.

Hypothesis $u_{3}$ was tested by checking whether the mean value of the classification accuracy, i.e., calculated from all $A_{i}$ values, is greater than 0.6.

## 6. Results

The following sections present the results obtained from studies 1 and 2 according to the previously described analyses and procedures.

### 6.1. Study 1

Table 6 presents the mean values of the resulting classification metrics for accuracy, precision, recall and F1 score, calculated and analyzed according to the procedures described in Section 5.1. Regarding the accuracy metric, the highest mean value achieved was 62.3% in test MULTI_G, whose model used a combination of facial and HR features. The HR feature in that case was calculated from the physical sensor, not remotely estimated. The second and third highest accuracy rates were 60.8% in test HR_G (HR from physical sensor only) and 60.4% in test MULTI_R (facial and remotely-estimared HR features), respectively. The highest values achieved for precision, recall and F1 score were 65.6%, 62.4%, and 58.1%, respectively, all in test HR_G.

Table 7 presents the minimum and maximum mean values for the resulting classification metrics. At least one subject in test MULTI_G has been classified with a mean accuracy of 98%, the highest value for that metric in all the tests. The worst mean accuracy value was 19% for at least one subject in test MULTI_R. Regarding precision, the highest mean value was 97% in test MULTI_G. In all tests but HR_G, at least one subject has been classified with zero precision (all the samples were classified wrongly). Regarding precision, the highest and lowest mean values were 98% and 12% in tests MULTI_G and FACE, respectively. Finally, regarding the F1 score, the highest mean value was 98% for at least one subject in test MULTI_G. All the tests presented zero as the lowest F1 score.

Finally, there are indications that a multifactorial model, which uses a combination of facial and HR features, performs with greater accuracy than a model that uses either facial or HR features. A Wilcoxon Signed Ranks test indicates that the mean accuracy was greater for a multifactorial model that uses facial and remotely estimated HR features, i.e., MULTI_R, than for a model that uses only remotely estimated HR, i.e., HR_R, Z=−2.00, p=0.044, r=0.26. However, there are no indications that the mean accuracy of such a multifactorial model, i.e., MULTI_R, is statistically significantly greater than the mean accuracy of a model that uses only facial features, i.e., FACE, Z=−1.10, p=0.267, r=0.14.

### 6.2. Study 2

#### 6.2.1. Self-reported emotional state

The emotional state of the subjects during the interaction with the Infinite Mario game is an important element of the experiment. Table 8 shows the mean value and standard deviation of the answers given in the self-reported emotional state questionnaire after each level of Infinite Mario.

Levels $A_{3}$ and $B_{3}$ presented 2.9 and 3.0 as the mean value for reported stress, respectively, the two highest mean values for stress. Using level $A_{1}$ as a baseline, since it presented the lowest median values for stress and boredom, i.e., 1.0 and 2.0 respectively, a Wilcoxon Signed-ranks test indicated different stress levels between $A_{1}$ (median 1.0) and $A_{3}$ (median 3.0), Z=−5.78, p<0.001, r=0.73. The same test also indicated different stress levels between $A_{1}$ (median 1.0) and $B_{3}$ (median 3.0), Z=−5.55, p<0.001, r=0.70.

Levels $B_{1}$ and $C_{1}$ presented 3.9 and 4.0 as the mean values for reported boredom, respectively, the two highest mean values for boredom. Repeating the use of level $A_{1}$ as a baseline, a Wilcoxon Signed-ranks test indicated different boredom levels between $A_{1}$ (median 2.0) and $B_{1}$ (median 4.0), Z=−6.14, p<0.001, r=0.77. The same test also indicated different boredom levels between $A_{1}$ (median 2.0) and $C_{1}$ (median 4.0), Z=−6.21, p<0.001, r=0.78.

As mentioned in Section 4.5.2, levels $A_{3}$ and $B_{3}$ were adjusted to be perceived as more stressful. Similarly, levels $B_{1}$ and $C_{1}$ were adjusted to be perceived as more boring. The results confirm with statistical significance that the adjustments applied to these levels of Infinite Mario indeed caused a particular emotional state. Even though the analysis presented here was performed on a group basis and the levels of Infinite Mario used in the evaluation of the method were selected on a user basis (according to individually self-reported emotional states), it is possible to conclude that there are indeed levels that were perceived with different emotional states. This is essential for the evaluation and validation of the proposed method.

#### 6.2.2. Emotion Classification

A subject’s emotional state during the interaction with particular levels of Infinite Mario was classified as stress or boredom using our proposed method. Table 9 presents the mean value of the resulting classification metrics for accuracy, precision, recall and F1 score, calculated and analyzed according to the procedures described in Section 5.2.

The proposed method was able to identify the emotional state of subjects with a mean accuracy of 61.6%. As previously mentioned, hypothesis $u_{3}$ states that a user-tailored model, i.e., neural network, trained on data samples from three calibration games of a given subject $S_{a}$, i.e., Mushroom, Platformer and Tetris, is able to classify the emotional state of samples extracted from an evaluation game, i.e., Infinite Mario, played by that same subject $S_{a}$ with a mean accuracy greater than 60% (calculated chance-level rate). A mean accuracy of 61.6% refutes the null hypothesis, supporting the claim of hypothesis $u_{3}$. It confirms the feasibility of the proposed method to perform better than chance-level estimations.

**Figure 12:**
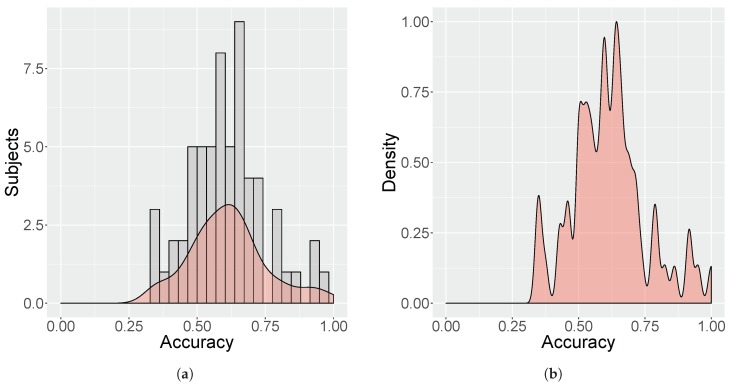
Distribution of accuracy values at a subject level. (**a**) histogram showing the number of subjects and the accuracy rate obtained in their emotion classification; (**b**) density curve regarding the distribution of accuracy values.

Since the subjects were evaluated independently, the mean classification accuracy is not enough to contextualize the estimations at a user level. Figure 12 provides a better context of the accuracy values distribution at a subject level, as demonstrated by a histogram and density curves. As illustrated in the histogram of Figure 12a, the majority of the subjects presented an emotion classification accuracy close to 60%. Particularly inaccurate estimations can be seen in a group of 13 subjects (20.9%) that were classified with a mean accuracy less than 50%. In contrast, a group of 14 subjects (22.5%) presented particularly precise emotion estimations with a classification accuracy greater than 75%.

Figure 12b shows a density curve with default bandwidth and an adjustment of 0.25 regarding the distribution of accuracy values. In this illustration, the area under any part of the curve provides the probability that the accuracy value would equal that particular group of samples. As expected, there is a greater likelihood that samples are classified with an accuracy of 60% (mean accuracy). Interestingly, there is the likelihood that samples are classified with an accuracy of 80% or 90%, which is more likely than a classification accuracy of 40%.

## 7. Discussion

### 7.1. Study 1

The results indicate that the application of a user-tailored model to remotely estimate the emotional state of players from videos of gaming sessions is feasible. A user-tailored neural network trained on data samples from two calibration games of a given subject $S_{i}$ is able to classify samples from a third calibration game of that same subject $S_{i}$. Such a model was tested in two configurations: MULTI_R and MULTI_G. Model MULTI_R, which uses a multifactorial feature set composed of facial and remotely estimated HR information, presented a mean classification accuracy of 60.4%. Model MULTI_G, which also uses a multifactorial feature set but differs in the acquisition of HR data, i.e., physical sensor instead of remote estimation, presented a mean classification accuracy of 62.3%. The slightly greater classification accuracy of model MULTI_G compared to MULTI_R suggests that more precise rPPG estimations of the HR could improve the overall classification accuracy of model MULTI_R. A Wilcoxon Signed Ranks test, however, has no statistically significant indication that the accuracy was greater for MULTI_G than for MULTI_R, Z=−1.76, p=0.078, r=0.23. Despite not being statistically significant, values p=0.078 and r=0.23 (small effect according to Cohen’s classification of effect size) suggest a trend towards that reasoning.

Hypothesis $u_{1}$ states that a user-tailored neural network using a multifactorial feature set performs with greater accuracy than a user-tailored neural network using facial features only. The Wilcoxon Signed Ranks test, mentioned in the previous section, presented no statistically significant indications that the classification accuracy of MULTI_R is greater than FACE. It is possible to speculate that a set of facial features, e.g., eyebrow and mouth analysis, has a greater potential to differentiate emotional states in a classifier. In particular, it might perform better than a classifier based on rPPG-estimated HR alone. Hypothesis $u_{2}$ states that a user-tailored neural network using a multifactorial feature set performs with greater accuracy than a user-tailored neural network using HR features only. The Wilcoxon Signed Ranks test, mentioned in the previous section, presents statistically significant indications that the classification accuracy of MULTI_R is greater than HR_R. It supports the claim of hypothesis $u_{2}$, confirming that a multifactorial model performs better than one based solely on remotely estimated HR data. The lower classification potential of remotely estimated HR, however, could be attributed to errors in the rPPG estimation process caused by noise, e.g., natural movement of subjects. As a consequence, a more precise HR estimation used in a multifactorial model allegedly contributes to producing a better classifier. A Wilcoxon Signed Ranks test confirms with statistical significance that the classification accuracy of MULTI_G, i.e., facial and HR from sensor, is greater than the accuracy of FACE, i.e., facial information only, Z=−2.12, p=0.033, r=0.28. It supports the previously mentioned idea that a precise HR estimation (from a physical sensor in the case of MULTI_G) combined with facial information is a better classifier than one using facial information alone, i.e., model FACE. Finally, a Wilcoxon Signed Ranks test does not indicate that the accuracy of MULTI_G is greater than HR_G, Z=−1.08, p=0.278, r=0.14. Consequentially, it seems that precise estimations of HR are important, but HR or facial information used separately is likely to be less important for classification than their joint, multifactorial use in the emotion classification process.

Overall, the mean accuracy rate of models MULTI_R and MULTI_G are 60.4% and 62.3%, respectively. The previously mentioned Wilcoxon Signed Ranks test does not indicate that the accuracy was greater for MULTI_G than for MULTI_R, so their performance could be the same. It is also important to stress the use of Leave One Session Out Cross Validation in the evaluation of each subject. It uses a completely independent and different game as the sampling source for the evaluation of each model, which strengthens the evaluation process. The reduced number of subjects in the study, i.e., 19, as well as samples used in the evaluation of each model, i.e., 38 on average, are in fact limiting factors. The reported results, however, provide insights into the feasibility of a multifactorial remote approach for emotion classification. Within the context of Study 1, our results suggest that a user-tailored neural network, based on remotely acquired data from video recordings, is able to classify emotional states. The analysis of different feature sets used for emotion classification suggests that precise estimations of HR are important. However, HR or facial information used separately is likely to be less important for classification than their combined use in a multifactorial emotion classification model based on remotely acquired data. The analysis performed in this study supports further research on a user-tailored model to remotely estimate the emotional states of players.

### 7.2. Study 2

The results of the proposed method for emotion detection based on remotely acquired signals show that the mean classification accuracy of 61.6% is better than a calculated 60% chance-level rate. As described in Section 5.2, the evaluation of the method has been performed as a balanced two-class problem with a sample size of n=64 (on average). In particular, the chance-level mean classification accuracy rate of 60% has been found by assuming a binomial distribution for the classification error to ensure a statistically significant classification [112]. The achieved mean classification accuracy of 61.6% supports the proposed hypothesis $u_{3}$, proving that a user-tailored model trained on data samples from three calibration games of a given subject is able to classify the emotional state of samples extracted from an evaluation game played by that same subject. The use of calibration games as emotion elicitation for training an emotion classifier is a novel aspect of the method presented here. The results support such an idea, showing that calibration games could be used as emotion elicitation material.

The mean classification accuracy of 61.6% is better than the calculated chance-level accuracy of 60%, and it outperforms previous work based on games and physical sensors whose classification accuracy varies between 48% and 55%, e.g., Chanel et al. [100]. Despite the results, our classification accuracy is still below the mean classification accuracy achieved in other affective computing studies. A survey conducted by Moghimi et al. [58] of over 33 affective computing studies undertaken since 1993 shows a mean classification accuracy of 77.91% (±12.76%, minimum of 50%, i.e., random classification, and maximum of 96.5%). The method proposed here is within such a reported range, however, a fair comparison of evaluation metrics is virtually impossible considering the different methods, setups and aims. For instance, the mentioned survey presents only six studies (18%) that used game-related stimuli; however, all of them used physical sensors to acquire user signals. Compared in isolation, the mean accuracy is a simple metric that can be used to estimate how well an approach can classify emotional states. However, the evaluation method and the procedures used for training/testing the model can profoundly influence accuracy results. For instance, Kukolja et al. [7] classify five emotions using kNN (nearest neighbors) based on physiological signals obtained with physical sensors. When Leave-One-Out Cross-Validation (LOOCV) is employed, i.e., available data are divided into parts and one part is left out while the rest is used for training, the mean evaluation accuracy is 78.76%. When LOSOCV is used, i.e., one experimental session is left out for testing and the remaining ones are used for training, the mean evaluation accuracy drops to 56.18%. Studies using LOOCV usually report mean classification accuracy in the range of 60–80%; however, LOOCV is less likely to be encountered in a real world situation. When the data available are divided into two groups, e.g., training and testing datasets, samples that are highly correlated, i.e., samples from the same game or session, could exist in both datasets. In the present experiment, for instance, data samples from the game being evaluated, i.e., Infinite Mario, are not in the training dataset. They are, in fact, used exclusively in the testing/evaluation dataset, which is completely independent from the training data. Classification performance on fresh data from the validation set is a better measure for how well the classifiers generalize [44]. Therefore, the use of LOOCV, even when k-fold cross-validation is used, presents testing samples that are considerably similar to those found in the training dataset, which could lead to higher mean classification accuracy.

It is also important to highlight how the signals used in the emotion classification are acquired. In the method proposed in the present experiment, all of the information used to build the user-tailored model is collected remotely, in a non-obtrusive manner. The previously mentioned mean classification accuracy of 77.91% from other affective computing studies depends on physical sensors to acquire a subject’s signals. A completely remote, emotion estimation setup presents a significant set of challenges. The results of the completely remote data acquisition employed by previous studies show an accuracy rate of 89% for negative, 56% for neutral and 78% for positive state identification [102]. For only stress detection, the mean classification accuracy reached best marks of 80% and 90% in contexts involving interactions with stressful images and the Stroop test [33]. Finally, in a context involving the detection of cognitive stress, the mean accuracy classification of rest vs. cognitive load has been reported as 86% [24]. All those studies rely on a completely remote method for data acquisition; however, the context of the classification is not focused on games research or in the use of games as the source of emotion elicitation for the training of a model. In the majority of cases, the subjects are also instructed to remain still, which is unlikely to happen in the natural interaction with games. Additionally, LOOCV or equivalent is used in some cases, which influences the model accuracy, as previously mentioned. As detailed in the survey by Moghimi et al. [58], there are many different experimental setups and approaches for affective computing. Given the peculiarities of each approach, including how the model is trained and evaluated, it would be unfair and naive to make a direct comparison of the studies. Attention should focus on the method, aims and evaluation of any presented approach, so merits can be decided.

Finally, an important factor in the present experiment is the material used to produce the emotional stimuli. In contrast to previous studies, the subjects interacted with complete digital games, not images, videos or text as content, to produce the emotional stimuli. The evaluation game, and the calibration games used in the experiment, are not gamified cognitive tests, e.g., Stroop test [101]. It strengthens the applicability of the results in the field of games research, which is the foundation and the aim of the proposed approach. Another remark is that a mean classification accuracy of 61.6% might not necessarily be connected to flaws in the proposed approach, but due to limitations in the labeling of ground data. The most reliable technique to assess and label emotional experiences in order to perform appropriate psycho-physiological signal classification is self-assessment of the emotional state [58]. However, even if a subject reported a particular level of Infinite Mario as stressful, it does not mean that all the samples collected from that level represent an emotional state of stress. It is plausible to believe that the subjects experienced fluctuations of emotions during a single level, e.g., stress, happiness, and even boredom. Such nuances are not captured by the labeling process used to create the evaluation dataset, which could lead to lower classification accuracy. The heterogeneous nature of the subject population, however, should ensure that such a factor is accounted for. It should be noted that the considerable number of subjects in the experiment, i.e., n=62, is greater than the average number of participants in previous affective computing studies, which is n=25.5 subjects per experiment [58]. This allows a broad evaluation of the proposed approach, accounting for different player profiles and supporting the claims of the previously mentioned hypothesis.

## 8. Limitations, Critique and Ethical Considerations

### 8.1. Limitations of Our Approach

One potential limitation of the work presented in this article is the nature of the calibration games. Even though they serve the purpose of emotion elicitation materials, they were designed and developed as ordinary games. Along the process, several decisions were made concerning different aspects of each game, which inevitably affected the end result. These decisions had an impact, for instance, on the genre of each calibration game, as well as its graphical appearance and the level of complexity of the mechanics. A calibration game should induce a state of boredom at the beginning of the interaction, thus users should easily understand its mechanics in order to perceive the game as boring without a long exposure. It entails that the game mechanics must be easily understandable, preferably without much text or tutorials. Users should not spend a considerable amount of time learning the game, otherwise the concepts that induce boredom might be misunderstood and the desired emotional state would not be induced. Additionally, all calibration games should not allow users to deliberately control the mechanics’ pace, since it was a key factor that was automatically controlled to induce stress towards the end of the session. Those constraints led the design of the calibration games towards more casual, 2D game mechanics. Even though games with similar characteristics exist, the proposed calibration games lack 3D content or a more complex interaction similar to those found in AAA COTS games, for instance. The genre/mechanics selected for the calibration games likely hinder several other genres and mechanics that could potentially be used as calibration games as well. The nature of the calibration games presented in this article does not cover the wide range of possible game types that exists, which limits its reach.

In that light, it could be argued that the calibration games proposed in this research only induced emotions elicited from the specific genres/mechanics that were selected. The use of 2D, casual foundations for the calibration games, could have conveyed a message of “old games” to a segment of subjects/users, which would likely impact their emotional reactions. On the other hand, the use of a more complex 3D game with sophisticated mechanics, e.g., Counter Strike, is likely to require a certain level of gaming skills from participants. In such a case, it would impact the interactions of subjects that are not very familiar with gaming, which was the case for some participants in the heterogeneous groups presented in this research. As mentioned previously, individuals have different cultural views and expectations; thus, a more complex game would make it even harder to balance the design of a game with its intended purpose of inducing boredom and stress. The game Infinite Mario was used in the validation process of the proposed method mainly due to its characteristics, e.g., easy to understand and play. Additionally, it allowed more control over the content generation associated with its mechanics; therefore, boring and stressful levels could be easily developed for the experiment mentioned in the article. It is plausible that the emotion classification results obtained with Infinite Mario could be generalized to similar games, especially since Super Mario influenced a range of platformer games. However, as previously mentioned, the use of another 2D, casual game for the validation could limit the generalization of the results.

Another limitation of this research concerns the accuracy obtained by the method in the classification of emotional states of boredom and stress. As presented in Section 6 and Section 7, the method achieved an accuracy of 61.6%. Even though this classification rate has statistical significance that proves the method performs better than random guessing, such a performance is still too low for commercial or even academic use. In its current state, the proposed method could not be used as the only tool to detect the emotional state of users, due to its noise. Additional measurements should accompany the proposed method to ensure a proper evaluation of the emotional context of subjects/users, e.g., questionnaires. However, the proposed method could still be used as an insight mechanism to analyze large amounts of video footage in an automated way, for instance. Despite the best efforts invested in this research to design an accurate emotion detector, the complexity of the task and the amount of man-power available limited the exploration process. Instead of aiming for a perfect tool, the research presented in this article focused on designing and rigorously evaluating each part of the proposed method. Such an approach is expected to eventually guide the construction of a more sophisticated emotion detector in the future.

It is important to highlight the technical limitations associated with the remote acquisition of physiological signals. The rPPG technique used in this research, as detailed in Section 3, is appropriate to deal with the natural behavior that users exhibit during their interaction with games. However, this technique was likely affected by other factors not scrutinized by the research in this article. For instance, the duration of the analysis window used for rPPG measurements of the HR has an impact on the results. The duration of the analysis window is debatable [18] and intrinsically related to the rPPG technique and hardware in use, such as the frame rate of the camera [113]. Another factor is that the setup of equipment used in both experiments rely on a dedicated light source. The use of controlled illumination aimed to reduce or mitigate noise in the rPPG estimations, potentially attributing any estimation errors to the interactions of the subjects, e.g., facial activity and body movement. Nonetheless, other factors probably could have impacted the accuracy, including the presence of glasses and the color of the skin. The results obtained with this research were achieved in a laboratory-like environment with controlled light source, which limits the generalization of the conclusions. As detailed in Section 3, a subject’s movement and changes in illumination are significant challenges to the estimation accuracy of rPPG techniques. The use of a controlled light source, however, was deemed necessary to concentrate efforts on the remote detection of the emotional state, not on the noise caused by different illumination patterns.

Another significant limitation is that the method proposed in this article can only detect two emotional states, i.e., stress and boredom. As detailed in Section 3, there are different models and theories about emotions. Previous works focused on emotion detection commonly classify the six basic emotions proposed by Ekman and Friesen [83], i.e., happiness, surprise, sadness, fear, anger and disgust. There are also a significant number of works that measure emotional states in terms of Russell’s Arousal–Valence space [93]. The method described in this article relies on the emotion elicitation provoked by the concept of calibration games, which are designed to be user-tailored materials that account for the differences among users in the emotion elicitation process. These games are, by design, limited to inducing only boredom and stress. It is plausible that other emotions are also elicited by the calibration games, e.g., happiness and anger. However, the very idea of constantly and endlessly increasing the difficulty of the games to account for the subjects’ individualities in the emotion elicitation process, e.g., different gaming skills and cultural expectations, directly limits the emotions that can be reasonably tracked without interrupting the gameplay. As demonstrated by the statistical tests performed in previous analysis [39], the emotional state of subjects is boredom at the beginning and stress at the end of the calibration games. Inducing a state of happiness in a player, for instance, is a complex task that depends on several components, including cultural factors and gaming preferences. The same reasoning can be extended to other emotions, such as anger, fear and disgust. Even for commercial games, significant resources are invested to ensure a game is properly balanced to be able to please a wider range of users; however, there is still no guarantee that this will happen. Consequentially, the focus put on detecting only boredom and stress in the method presented in this article is a constraint created to counterbalance the differences that exist among subjects, regarding their different gaming, cultural and emotional profiles. However, it is important to mention that emotional states of stress and boredom are still relevant to the industry or studies in HCI and game research. Flow theory is commonly used in game research to model players emotions, which is directly connected to stress and boredom. Finally, there are also a number of studies that cast doubt on the viability of static models of affect based on sensed physiological data, as our method claims to do. As the meta-analytic investigation conducted by Siegel et al. [114] demonstrates, the mapping between an emotion category and a specific ANS response pattern is unlikely to be a 1-to-1 relation. Consequentially, changes in the ANS during emotion reactions can be interpreted more accurately as a population of instances that are sensitive to context. Such reasoning might explain why our approach achieves recognition rates of 61%, as it is trying to model something that is not static in the way that a fingerprint is static, for instance. However, we still believe our results demonstrate the complexities of modelling emotional states, even if it could be argued that we are not modeling a category of emotional states because there is no 1-to-1 mapping, be it a facial or a HR response. If nothing else, our research and results serve as the basis to show the limitations of using game-elicited emotions to train a neural network to recognize and perform a 1-to-1 mapping of facial and HR responses into emotional states of stress and boredom within the context of games.

### 8.2. Ethical Considerations

Mason [115] mentions that the facts of an ethical situation can be summarized by four factors. The first is the identification of the moral agent, which is the one causing the technology-induced change. The second relates to the available courses of action that the moral agent can undertake. It is not always possible, or even viable, to choose more than one course of action. Consequentially, it must be selected according to the best interests of all parties involved. Additionally, a course of action is bound to have consequences, which can be irreversible. In that light, the third factor to emerge is the delimitation of the results that are expected to occur if each act is taken. A proper delimitation of results makes it clear for the involved parties how to measure the impact and implications of an act. Finally, the fourth factor is the identification of the stakeholders who will be affected by the consequences of the acts. One of the main goals of the technology developed in this research is a non-obtrusive form of emotion detection. Given that a person has agreed to have a user-tailored model of him/herself created, i.e., play the calibration games while being filmed, any moral agent, i.e., researcher or company, is then able to use such data freely and unrestrictedly. After the model has been trained, the person used to train said model can be indefinitely surveyed in a context of gaming. Once trained, the model can be easily transferred to another moral agent, e.g., another institution or company, and used at a later time. Even though the proposed method is constrained by a gaming context, it can still be widely used. If the person in question, who is the stakeholder of the process, is not properly and clearly informed about the identity of the moral agents and the delineation of the results expected from the use of his/her model, an ethical issue may exist.

An ethical issue is said to arise whenever one party in pursuit of its goals engages in behavior that materially affects the ability of another party to pursue its goals [115]. One could claim that sharing a person’s user-tailored model among institutions/companies is not materially affecting that person. Additionally, people are more prepared to accept potentially invasive technology if they consider that its benefits outweigh potential risks [116]. However, one of the moral agents might be a game development company that uses the model to detect the emotional state of a person, in order to maximize the sale of in-game goods. In that case, the act could materially affect the person, which would clearly be an ethical issue if the person was never made aware of such a possible use of his/her model. As previously mentioned, the facts of an ethical situation must be clear, otherwise obscure information about courses of action, delimitation of results and even the identity of the moral agents could lead stakeholders into making poor judgments regarding ethics and privacy.

Another implication of non-obtrusive technologies is how it influences the ability of a user to decline the propagation of any information. In the context of games research, for instance, if a subject answers a questionnaire about a game being played, it is completely plausible to assume that the subject could deliberately lie about the answers. Subjects might even decline to answer a particular question about emotions, if they feel uncomfortable, for instance. If the method proposed by this research is used to detect emotional states and one assumes that the subjects have previously agreed to the creation of a user-tailored model of themselves, then they do not have the option to decline to answer a query about emotions. A researcher might have a previously trained model of a subject, e.g., from an old experiment, which can be used again for the same subject, however in a different context. The method proposed in this research can be adapted to be trained on data from a group instead of an individual, i.e., group model instead of a user-tailored model. In that case, the trained model could be applied to any person (or subject), without the need for them to play the calibration games. It is plausible to believe that such a configuration of the method could be used by companies to survey a player’s emotional responses to a particular game. A company could, for instance, apply the method to online videos, e.g., “Let’s play” videos on YouTube, to gather unsolicited emotional data. If the videos are freely available, does it mean such use of the method is ethical? Was the person in the video thinking about having his/her emotions automatically detected by a software when he/she made the video?

Regarding pricavy and personal data, discussions in the field of human–computer interaction are common and there is a clear indication that HCI tools must not invade a user’s privacy [117]. Any tool’s capacity to monitor and concentrate information about somebody’s behavior must not be misused. The technology presented in this research significantly relates to privacy. As defined by Culnan [118], privacy is the ability of the individual to control the terms under which personal information is acquired and used. When a system collects personal information, which is the case of the method in this research, information privacy becomes an issue. Stone et al. [119] define information privacy as the ability of the individual to personally control information about one’s self. In the context of this research, information privacy relates to how a person controls the digital data collected from him/herself, e.g., video recordings and the user-tailored model. As previously mentioned, the technology presented in this research has moral and ethical implications, which leads to information privacy implications. Privacy is extremely contextual, based in the specifics of by whom, for what, where, why, and when a system is being used [120]. In that sense, individuals monitored and analyzed by the technology presented in this research might have divergent opinions regarding information privacy. Some individuals might believe the use of such technology is beneficial and could be used to enhance their gaming experience, for instance. On the other hand, some individuals might oppose the use of such technology, due to concerns about information privacy. Awad and Krishnan [121] show that consumers using online shopping websites who desire greater information transparency are less willing to be profiled. In contrast, users that do want a more personalized experience when shopping online are more willing to be profiled. One possible solution for such a problem, which is a recommendation by Awad and Krishnan [121], is the utilization of mechanisms that account for both types of clients, the ones willing to be profiled to increase service personalization and those that are not.

The method proposed in this research is based on the analysis of video recordings. Normally, users are not concerned about a video recording beyond the issue of the usage of their personal image. The present research, however, uses several techniques to collect additional data from those video recordings, including facial analysis and HR information. When being filmed during the interaction with a set of calibration games, a person might not be aware of the amount of information that is actually being collected. How such data are stored, processed and used is a matter of information privacy. As previously mentioned, people are more prepared to accept potentially invasive technology if they consider that its benefits outweigh potential risks [116]. Users constantly decide and account for the trade-off between the benefit of a solution and the privacy implications of such act. Nguyen et al. [122] show that, when privacy was evaluated against usability, convenience, and speed, the concern for privacy was relatively high. However, when compared to cost, concern for privacy was relatively low. This suggests that people have a clear trade-off between price/cost and privacy. As Awad and Krishnan [121] demonstrate, some consumers of online shopping are willing to give personal information in exchange for better services. Consequentially, users might be willing to be filmed and analyzed by a software, to have a personalized emotion detector to improve game experience, for instance, especially if they see benefits in any existing trade-off analysis taking place. What needs to be clear to those users, however, is the kind of data being collected, by whom and how it will be used.

Technology affects privacy because it changes the control over personal data that users have [123]. The technology presented in this research, if used in misleading ways, contributes to reducing the control people have over personal data. Ensuring users know what is happening, which can be achieved with clear information practices, is paramount. Langheinrich [124] reveals the principles that guide system design based on privacy legislation in use. The principles of notice, choice and consent mentioned by the author are essential to the technology presented here. Making users notice what is happening and what is being collected, as well as allowing choice and consent of the process, is the bare minimum to ensure privacy.

## 9. Conclusions

This paper presents a method for the remote detection of emotions during the interaction with games. The method works by training an emotion classifier using remotely extracted psychophysiological signals, i.e., HR and facial actions, from users playing game-based emotion elicitation materials. Such materials, named calibration games, are carefully designed games that induce emotional states of boredom and stress on a user-tailored basis, instead of a group perspective. It accounts for differences users might have regarding gaming skills and expectations during the emotion elicitation. The emotion classifier is also trained as a user-tailored neural network, i.e., data from a given user are employed to train a single neural network tailored to that given user. Finally, after the training process, the signals that a given user exhibits during the interaction with an ordinary, non-calibration game are remotely extracted and fed into the previously trained neural network. The neural network then outputs the emotional state of that given user in that particular game. The novelty of our approach relies on (a) user-tailored readings as opposed to the commonly used group approach, (b) the use of custom-designed, game-based emotion elicitation material that provokes emotional changes used to train an emotion classifier, along with (c) the use of facial analysis and rPPG to extract the psychophysiological signals involved in the process.

We presented a systematic evaluation regarding the feasibility and accuracy of our proposed method in two distinct experiments. The first experiment tested a user-tailored neural network trained on data samples from two calibration games of a given subject, which was then used to classify samples from a third calibration game of that same subject. The evaluation included the testing of different user signals, such as HR and facial data combined or used separately. The intent of such an evaluation was to better understand the benefits of a multifactorial analysis, confirming that it indeed produces better estimations of the emotional state of users. The experiment was limited by the number of games available for analysis, i.e., three, and the reduced number of subjects. Despite these constraints, the results suggest the feasibility of our non-obtrusive, game-based and user-tailored emotion detector. In order to further evaluate our method, a final validation was conducted in the second experiment with a larger sample size, i.e., n=62. In this experiment, the three previously mentioned calibration games were used as emotion elicitation materials to train a user-tailored model, i.e., neural network. This model was then used to detect the emotional state of each user during the interaction with a fourth game, i.e., Infinite Mario. Some levels of Infinite Mario were adjusted so that subjects would more likely perceive them as stressful or boring, thus allowing the proposed method to be evaluated according to such differences in the emotional state. The analysis performed on the levels of Infinite Mario has shown with statistical significance that they were indeed perceived as stressful and boring, which should be detected by our method. Following the expectations inferred by the first experiment, the proposed method was able to identify emotional states of stress and boredom of subjects playing Infinite Mario with a mean accuracy of 61.6%. The results confirm with statistical significance that the proposed method indeed classifies emotional states, achieving an accuracy rate better than chance-level classification. It supports our initially mentioned overarching hypothesis that an emotion detection approach that uses remotely acquired signals, i.e., HR and FA, as input of a machine learning model, i.e., neural network that is trained on a user-tailored basis (one subject produces one model) using calibration games as emotion elicitation can achieve an accuracy rate better than chance-level classification.

Finally, it is important to highlight that our proposed method for emotion detection based on calibration games, remote sensing and a user-tailored multifactorial analysis has not been found in the literature. Our experimental setup featured significant challenges not faced by previous works, such as the use of game-based emotion elicitation and players not being instructed regarding how to behave during their interaction with the games, i.e., natural behavior. That configuration presents a set of unique challenges that inherently affect the results of an emotion detection procedure, particularly when the process uses remote estimation of psychophysiological signals. Compared to existing works in the literature, the mean classification accuracy of 61.6% achieved by our proposed method is still below the mean classification accuracy achieved by other affective computing studies, i.e., 77.9%. A fair comparison of these numbers, however, is not possible. Each study is conducted in particular situations, using different emotion elicitation materials and different training/testing models. Our method focuses on the individual, not the group, which is a common factor found in the literature. Another important and highly distinctive difference between the present work and existing ones is how data are obtained to train and evaluate the emotion detection model. Our method uses a completely independent dataset to train the model, which is obtained from the natural interactions of users with game-focused elicitation materials, i.e., calibration games. These games are similar to COTS games, which portray a more real gaming experience. The evaluation of the method was conducted on the Infinite Mario game, which mimics the commercial Super Mario game. Data from this game were never used in the trained model, yet it was able to classify the emotional state of users. The evaluation of classification performance on fresh data are a better measure of how well classifiers generalize [44]. Several works focused on emotion detection do not use game-focused materials for training of a model. Commonly they evaluate accuracy by testings samples that are considerably similar to those found in the training dataset, e.g., splitting available data into training and evaluation datasets, which is different from what is presented in this paper.

Our proposed method for the remote detection of emotions has been conceived on the basis of established theories and it has been carefully evaluated in experimental setups. The process of detecting user emotions is a complex task that involves theories and contributions from different fields. The results presented in this paper prove our method is feasible, however limited regarding accuracy. We stress that the primary context where our approach is likely to be used is not clinical analysis, where high accuracy is a requirement. Instead, our primary usage context is game development and games research, where our tool can help researchers and practitioners to obtain information about user emotions in an automated way. Such usage is possibly performed on medium to large scale in the case of companies, and the main goal is not to disturb or interrupt players. Nevertheless, our proposed approach is a solid initiative to move away from questionnaires and physical sensors into a non-obtrusive, remote-based solution for detecting emotions in a context involving more naturalistic behaviour of users during their interaction with games. As future work, we aim to further investigate machine learning models able to improve the accuracy of our emotion classifier, including the enhancement of existing and possible exploration of new features for the model. Additionally, we intend to test different games for both the calibration and the testing phases. Particularly to the testing phase, commercial games capable of inducing levels of stress and anxiety such as Resident Evil, Silent Hill, or Dead Space are suitable candidates for future analysis.

## Figures and Tables

**Table 1:** Description of features used for classification.

Notation	Name	Description
$F_{1}$	Mouth outer	Monitor the zygomatic muscle.
$F_{2}$	Mouth corner	Monitor the zygomatic muscle.
$F_{3}$	Eye area	Monitor the orbicularis oculi muscle, e.g., blinking.
$F_{4}$	Eyebrow activity	Monitor the corrugator muscle.
$F_{5}$	Face area	Monitor facial movement to and away from the camera
$F_{6}$	Face motion	Describe the total distance the head has moved in any direction in a short period of time.
$F_{7}$	Facial COM	Describe the overall movement of all 68 facial landmarks.
$F_{8}$	Remote HR	HR estimated using the rPPG technique proposed by Poh et al. [45].

**Table 2:** Self-reported levels of skill at playing video games for subjects in Study 1 (n=20) and Study 2 (n=62).

Answer	Subjects in Study 1 (%)	Subjects in Study 2 (%)
No skill	1 (5%)	6 (9.7%)
Not very skilled	10 (50%)	19 (30.6%)
Moderately skilled	7 (35%)	25 (40.3%)
Very skilled	2 (10%)	12 (19.9%)

**Table 3:** Self-reported number of hours per week subjects in Study 1 (n=20) and Study 2 (n=62) played any type of video game over the last year.

Answer (in Hours)	Subjects in Study 1 (%)	Subjects in Study 2 (%)
More than 10	2 (10%)	25 (40.3%)
5 to 10	6 (30%)	7 (11.3%)
3 to 4	2 (10%)	6 (9.7%)
1 to 3	2 (10%)	5 (8.1%)
0 to 1	4 (20%)	10 (16.1%)
0	4 (20%)	9 (14.5%)

**Table 4:** Levels of Infinite Mario and adjustments made to induce a particular emotional state.

Level	Type	Emotion	Adjustments
$A_{1}$	Overground	Any	Reduced number of interactable/collectable items and terrain obstacles; no power-ups; only 2 enemies and 1 gap (with width of Mario himself); Mario starts in big state.
$A_{2}$	Underground	Any	Regular number of interactable/collectable items, terrain obstacles, power-ups and enemies. Mario starts in small state.
$A_{3}$	Castle	Stress	Several gaps (with varying widths); reduced number of interactable items; no collectables/power-ups; several enemies; reduced time to complete level. Mario remains in small state. Mario starts with 5 lives. Available level time is 80 s.
$B_{1}$	Overground	Boredom	Auto-scrolling camera; reduced number of interactable/collectable items; few terrain obstacles; no gaps, power-ups, or enemies. Mario remains in big state.
$B_{2}$	Underground	Any	Regular number of interactable/collectable items, terrain obstacles, power-ups and enemies. Mario starts in small state.
$B_{3}$	Castle	Stress	Several gaps (with varying widths); reduced number of interactable items; no collectables/power-ups; several enemies; reduced time to complete level. Mario remains in small state. Mario starts with 5 lives. Available level time is 80 s.
$C_{1}$	Overground	Boredom	Auto-scrolling camera; reduced number of interactable/collectable items; few terrain obstacles; no gaps, power-ups, or enemies. Mario remains in big state.

**Table 5:** Tests and their respective feature sets.

$T_{i}$	Name	Feature Set	Note
1	MULTI_R	$F_{1}$, $F_{2}$, $F_{3}$, $F_{4}$, $F_{5}$, $F_{6}$, $F_{8}$	Facial analysis, rPPG-estimated HR.
2	MULTI_G	$F_{1}$, $F_{2}$, $F_{3}$, $F_{4}$, $F_{5}$, $F_{6}$, $F_{9}$	Facial analysis, HR from physical sensor.
3	FACE	$F_{1}$, $F_{2}$, $F_{3}$, $F_{4}$, $F_{5}$, $F_{6}$	Facial analysis only.
4	HR_R	$F_{8}$	rPPG-estimated HR only.
5	HR_G	$F_{9}$	HR from physical sensor only.

**Table 6:** Mean values of resulting classification metrics (Study 1).

Test	Accuracy	Precision	Recall	F1
MULTI_R	0.604	0.612	0.599	0.521
MULTI_G	0.623	0.583	0.607	0.514
FACE	0.594	0.601	0.585	0.507
HR_R	0.547	0.541	0.545	0.497
HR_G	0.608	0.656	0.624	0.581

**Table 7:** Minimum and maximum mean values of resulting classification metrics (Study 1).

Test	Accuracy		Precision		Recall		F1	
	min	max	min	max	min	max	min	max
MULTI_R	0.19	0.91	0.00	0.95	0.19	0.87	0.00	0.91
MULTI_G	0.25	0.98	0.00	0.97	0.13	0.98	0.00	0.98
FACE	0.26	0.90	0.00	0.95	0.12	0.90	0.00	0.89
HR_R	0.36	0.72	0.00	0.79	0.18	0.77	0.00	0.67
HR_G	0.38	0.82	0.26	0.85	0.23	0.87	0.00	0.81

**Table 8:** Mean value of the answers given in the self-reported emotional state questionnaire after levels of Infinite Mario (Study 2).

Level	Stress	Boredom
$A_{1}$	1.6 ± 0.8	2.3 ± 1.2
$A_{2}$	2.1 ± 0.9	1.8 ± 1.1
$A_{3}$	2.9 ± 0.9	1.9 ± 1.2
$B_{1}$	1.5 ± 1.0	3.9 ± 1.2
$B_{2}$	2.0 ± 0.8	2.2 ± 1.2
$B_{3}$	3.0 ± 1.1	2.1 ± 1.2
$C_{1}$	1.3 ± 0.7	4.0 ± 1.2

**Table 9:** Mean values of resulting classification metrics (Study 2).

Accuracy	Precision	Recall	F1
0.6158	0.6163	0.6127	0.5786

## Abbreviations

The following abbreviations are used in this manuscript:

ANS	Autonomic Nervous System
AUC	Area Under the Curve
BSS	Blind Source Separation
BVP	Blood Volume Pulse
CLNF	Constrained Local Neural Fields
CMA	Circumplex Model of Affect
COTS	Commercial Off-the-shelf
DSR	Design Science Research
ECG	Electrocardiogram
EEG	Electroencephalogram
EMG	Electromyography
FA	Facial Actions
FACS	Facial Action Coding System
FPS	Frames Per Second
HCI	Human–Computer Interaction
HR	Heart Rate
HRV	Hear Rate Variability
ICA	Independent Component Analysis
LOOCV	Leave-One-Out Cross-Validation
LOSOCV	Leave-One-Session-Out Cross-Validation
PNS	Parasympathetic Nervous System
RGB	Red Green Blue
ROI	Region of Interest
rPPG	Remote Photoplethysmography
RR	Respiratory Rate
SNS	Sympathetic Nervous System
SVM	Support Vector Machine
